# RNA-Seq for Enrichment and Analysis of *IRF5* Transcript Expression in SLE

**DOI:** 10.1371/journal.pone.0054487

**Published:** 2013-01-18

**Authors:** Rivka C. Stone, Peicheng Du, Di Feng, Kopal Dhawan, Lars Rönnblom, Maija-Leena Eloranta, Robert Donnelly, Betsy J. Barnes

**Affiliations:** 1 Department of Biochemistry & Molecular Biology, New Jersey Medical School, UMDNJ, Newark, New Jersey, United States of America; 2 New Jersey Medical School-University Hospital Cancer Center, UMDNJ, Newark, New Jersey, United States of America; 3 High Performance and Research Computing, Department of Information Systems and Technology, UMDNJ, Newark, New Jersey, United States of America; 4 Molecular Resource Facility, New Jersey Medical School, UMDNJ, Newark, New Jersey, United States of America; 5 Department of Medical Sciences, Section of Rheumatology, Uppsala University, Uppsala, Sweden; Beth Israel Deaconess Medical Center, United States of America

## Abstract

Polymorphisms in the interferon regulatory factor 5 (*IRF5*) gene have been consistently replicated and shown to confer risk for or protection from the development of systemic lupus erythematosus (SLE). IRF5 expression is significantly upregulated in SLE patients and upregulation associates with *IRF5*-SLE risk haplotypes. *IRF5* alternative splicing has also been shown to be elevated in SLE patients. Given that human *IRF5* exists as multiple alternatively spliced transcripts with distinct function(s), it is important to determine whether the *IRF5* transcript profile expressed in healthy donor immune cells is different from that expressed in SLE patients. Moreover, it is not currently known whether an *IRF5*-SLE risk haplotype defines the profile of *IRF5* transcripts expressed. Using standard molecular cloning techniques, we identified and isolated 14 new differentially spliced *IRF5* transcript variants from purified monocytes of healthy donors and SLE patients to generate an *IRF5* variant transcriptome. Next-generation sequencing was then used to perform in-depth and quantitative analysis of full-length *IRF5* transcript expression in primary immune cells of SLE patients and healthy donors by next-generation sequencing. Evidence for additional alternatively spliced transcripts was obtained from *de novo* junction discovery. Data from these studies support the overall complexity of *IRF5* alternative splicing in SLE. Results from next-generation sequencing correlated with cloning and gave similar abundance rankings in SLE patients thus supporting the use of this new technology for in-depth single gene transcript profiling. Results from this study provide the first proof that 1) SLE patients express an *IRF5* transcript signature that is distinct from healthy donors, 2) an *IRF5*-SLE risk haplotype defines the top four most abundant *IRF5* transcripts expressed in SLE patients, and 3) an *IRF5* transcript signature enables clustering of SLE patients with the H2 risk haplotype.

## Introduction

Systemic lupus erythematosus (SLE) is a complex systemic autoimmune disease characterized by elevated type I interferon (IFN) production, a break of immune tolerance to self-antigens, persistent production of pathogenic autoantibodies, complement activation, and immune complex (IC) deposition resulting in inflammation and end organ damage [1]. While the underlying etiology of SLE remains obscure, several lines of evidence document the importance of genetic factors [2]–[3]. One such factor, the interferon regulatory factor 5 (*IRF5*) gene, was identified in the susceptibility to develop SLE [4]. This initial finding, and the subsequent replication in numerous patient cohorts [4]–[10], marked an important break-through in our understanding of SLE pathogenesis given the critical role of IRF5 in regulating type I IFN expression and mediating Toll-like receptor (TLR) signaling [11]–[14]. Polymorphisms in the *IRF5* gene have been robustly associated with susceptibility to SLE in multiple populations with varying ethnic ancestries [4]–[10]. In each population, a distinct group of *IRF5* single nucleotide polymorphisms (SNPs) and genetic variants form haplotypes that confer risk for, or protection from, the development of SLE. It has recently been demonstrated that IRF5 expression and alternative splicing are significantly elevated in primary purified peripheral blood mononuclear cells (PBMC) from SLE patients; enhanced transcript and protein levels were associated with the *IRF5*-SLE H2 homozygous full risk haplotype in purified monocytes (Mo) [15]. The *IRF5*-SLE H2 haplotype consists of two copies of the 4xCGGGG promoter indel, the T-allele of SNP rs2004640, A-allele of SNP rs10954213, and C-allele of SNP rs10488631 [16]. Further studies have shown that *IRF5*-SLE risk haplotypes are associated with serum IFNα activity in SLE patients [17]–[18].

IRF5 is a member of the IRF family of transcription factors [11] and exists as multiple alternatively spliced 1.6-kb transcripts each encoding proteins with distinct cell type-specific expression, regulation and function [19]. IRF5 is a DNA binding protein primarily studied for its role in controlling inflammatory and immune responses, as well as mediating DNA damage- and death receptor-induced apoptosis [11]–[14], [19]–[29]. Given its important role(s) in host immunity, it is not surprising to find that it is constitutively expressed in cells of the immune system, particularly plasmacytoid dendritic cells (PDC), Mo, Mo-derived dendritic cells, and activated B cells [11], [19], [30].

Recent data in mice support a critical role for IRF5 in SLE pathogenesis. Consistent observations in murine models of lupus indicate that loss of *Irf5* protects mice from lupus-like disease [31]–[33]. In the pristane-induced model, *Irf5^−/−^* mice lack pathogenic autoantibodies and glomerular deposits as seen in their wild-type counterparts [32]. IRF5 is absolutely required for disease development in *FcγRIIB^−/−^Yaa* and *FcγRIIB^−/−^* mice [31] and Mrl/lpr *Irf5^−/−^* mice survived longer and had lower levels of autoantibodies as well as inflammatory cytokines and IFNα in their serum [33]. Additionally, autoimmune disease-prone NZB/W mice that spontaneously develop lupus-like disease were shown to display elevated constitutive levels of *Irf5* [34]. However, unlike human *IRF5* that exists as multiple alternatively spliced variants [19], murine *IRF5* appears to exist as a single major variant [28], suggesting inherent complexities between studies of IRF5 in human SLE and murine lupus-like disease; nevertheless, current data support a critical role for IRF5 in both human and murine SLE pathogenesis.

Results from genetic association studies have begun to shed light on the genetic risk conferred by an *IRF5* risk haplotype; however, to date, these studies have only been associative/correlative with other clinical manifestations of SLE, *e.g*. serum IFNα activity, autoantibody production and gene expression [18], [35]. One might speculate that if the IRF5 protein(s) encoded by the genetic variation is a mediator of such effects, then we should theoretically be able to detect this in the profile of *IRF5* transcripts expressed. Indeed, it has been hypothesized that an *IRF5*-SLE risk haplotype will confer changes in *IRF5* transcription, protein/isoform expression, and biological function [4], [7]–[8], [10], [36]; however, recent data from traditional cloning and sequencing suggests that haplotype does not alter the profile of transcripts expressed [8]. The aim of this study was to use a combination of molecular cloning and next-generation sequencing technologies to obtain sufficient coverage depth in primary immune cells of SLE patients and healthy donors to clearly determine 1) whether SLE patients express an *IRF5* transcript signature that is distinct from healthy donors, and 2) whether an *IRF5*-SLE risk haplotype determines the *IRF5* transcript signature, thus controlling and/or contributing to the global pathologic function(s) of IRF5 in SLE.

## Results

### Profiling IRF5 Transcript Expression by Traditional Molecular Cloning

**Figure 1:**
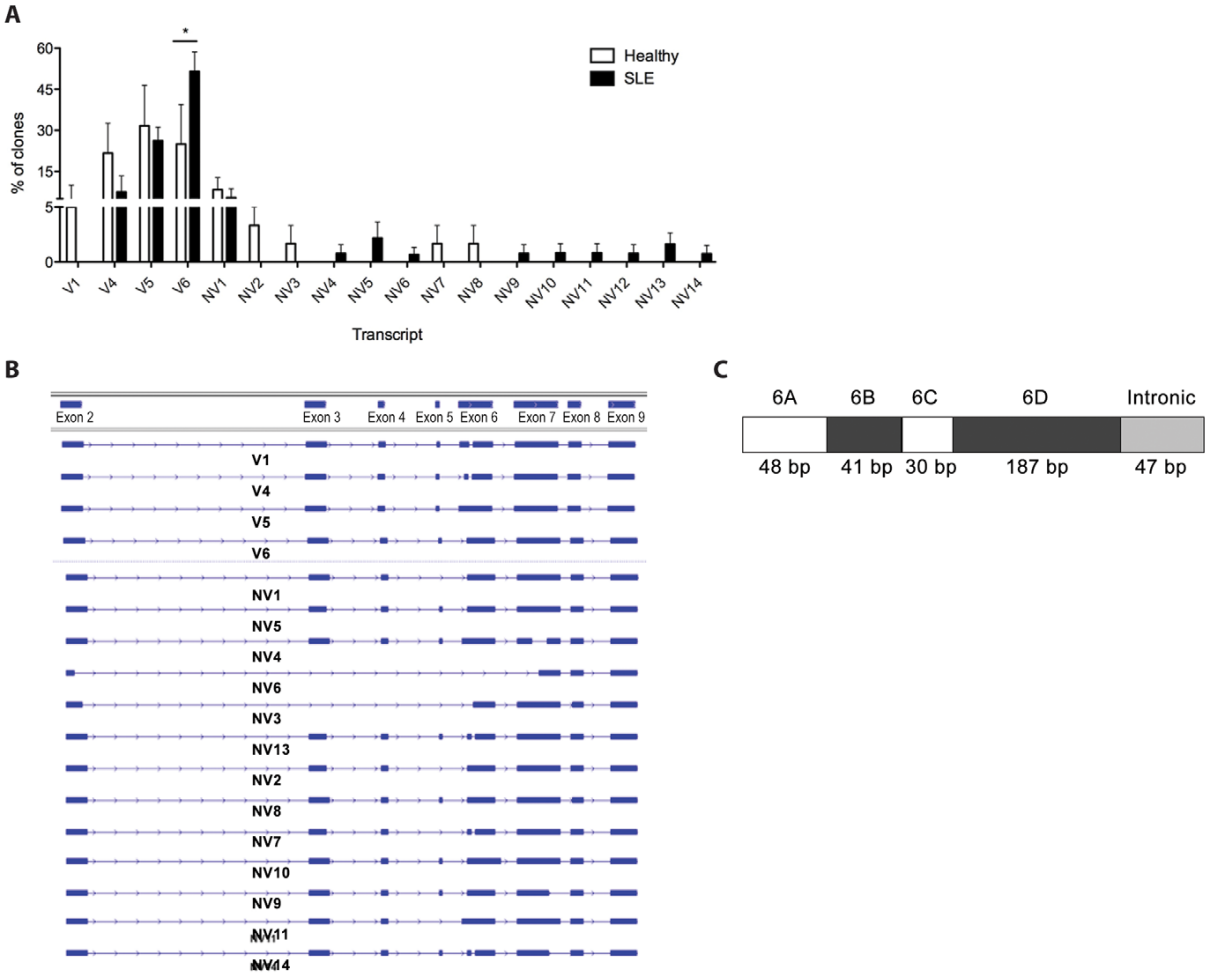
Identification of *IRF5* transcript variants expressed in immune cells of SLE patients and healthy donors. *A,* A profile of *IRF5* transcripts expressed in purified Mo of healthy donors (n = 3) and SLE patients (n = 6) was generated by molecular cloning and sequencing. PCR amplification was from non-coding Ex1a through Exon 9. At least 20 clones from each donor were obtained. Data are represented as a pooled % of total clones after calculating % of clones per variant per donor. Statistical analysis was performed using the two-way Anova with Bonferroni post-test. Known transcript variants V1, V4, V5 and V6 are depicted with novel variants (NV1-NV14). *B,* Scheme illustrating all *IRF5* transcripts cloned from purified Mo of healthy donors and SLE patients in a display generated using IGV software. Transcripts are comprised of exons (blue rectangles) spliced from the full *IRF5* genomic sequence (horizontal lines with arrowheads). Transcript NV12 is not shown since IGV software displays each transcript using the *IRF5* genomic sequence as a reference and is thus unable to represent duplications; NV12 contains a 12-bp internal duplication of a portion of exon 8 but is otherwise identical to transcript NV1. *C,* Diagram of exon 6 divided into portions 6A-6D as found in alternatively spliced *IRF5* transcripts. Ex6a and/or Ex6c (white rectangles) are deleted in several of the transcripts. The intronic insertion of transcript NV10 is shown as well.

Significant differences in *IRF5* expression levels have been detected in purified PBMC of healthy donors and SLE patients [7]–[8], [15]; yet, in general, this heterogeneous population of cells expresses low levels of total *IRF5* [19]. IRF5 expression has been shown to differ between immune cell subpopulations with Mo expressing the highest levels [19], [30], [37]. We have demonstrated a significant association between elevated IRF5 expression in primary Mo of SLE patients with the *IRF5*-SLE H2 risk haplotype [15]; in addition, we recently showed that IRF5 is constitutively activated in Mo, and not T or natural killer (NK) cells, from SLE patients compared to healthy donors [37]. Based on these data, we focused our expression profiling experiments on purified Mo from healthy donors and SLE patients. Given that new alternatively spliced *IRF5* transcript variants continue to be identified [7]–[8], [19], we initiated our study with the molecular cloning of full-length transcripts from this cell population. One-step RT-PCR was performed using primers that amplify all transcripts originating from the non-coding exon 1a (Ex1a) of *IRF5* [15], [19]. At least four non-coding alternative first exons (Ex1a, 1b, 1c and 1d) in the 5'-untranslated region (5'-UTR) of the *IRF5* gene have been identified [19]. We focused this study on transcripts that utilize Ex1a only since it has previously been shown that Ex1a transcripts are expressed at higher levels than other Ex1 transcripts [8], [19] and expression of known transcripts (V1, V4, V5 and V6) appears to be equivalent independent of Ex1 alternative utilization [8]. Indeed, cloning from Ex1a or Ex1c gave nearly identical expression of known *IRF5* variants V1, V2/V6, V3/V4 and V5 (data not shown). Results in Fig. 1A summarize the data illustrating the complete absence of V1 transcripts and significantly elevated V6 transcripts in SLE patients (n = 6) as compared to healthy donors (n = 3). Mo from both healthy donors and SLE patients shared predominant expression of known variants V4, V5 and V6, as well as the novel variant NV1. A total of five new *IRF5* variants were identified in Mo of healthy donors (Fig. 1A; NV1, NV2, NV3, NV7, NV8), four of which were exclusive to these healthy donors; nine new *IRF5* transcript variants were identified in the SLE patients examined.

A unique combination of insertions and deletions in coding exons 2–9 define the 14 new transcripts (NV1-NV14) isolated from purified Mo (Fig. 1B). Some of the new transcripts share deletions previously observed; the 30-nucleotide deletion seen in V1 and V4 is also seen in transcripts NV7, NV13 and NV14. Transcripts NV2, NV5, NV7 and NV13 share a common 28-nucleotide deletion at the end of exon 3 that has been described previously [7]. New deletions were observed as well; exon 5 is completely deleted in transcripts NV1, NV2, NV3, NV6, NV7 and NV11, and large internal deletions spanning more than 2 exons are seen in transcripts NV3 and NV6. Transcripts NV3 and NV8 share a common 19-nucleotide deletion in exon 8, and NV12 is identical to NV1 except it contains a duplication of 12 nucleotides in exon 8. Transcripts NV4, NV6, NV9 and NV14 have internal deletions in exon 7. Transcripts were also identified that contain combinations of insertions and deletions in exon 6, as diagrammed in Fig. 1C; exon 6A and 6C deletions were previously reported in known transcripts V1, V4, and V6 [19] while a novel 47-bp intronic insertion was identified in transcript NV10. Sequences of newly cloned transcripts are listed in Dataset S1.

### PCR Enrichment and Next-generation Sequencing

The power of standard molecular cloning to accurately profile *IRF5* expression in SLE patients and healthy donors is limited by the number of *IRF5*-positive clones analyzed; a large number of clones would be required to attain a statistically rigorous profile, requiring great resources and financial expenditure. Additionally, it has been observed that certain cDNAs cannot be obtained by molecular cloning techniques [38]; thus, a transcript profile obtained by cloning alone may be inaccurate. To overcome some of these issues, we utilized the power of next-generation sequencing to gain an unbiased view of *IRF5* transcript profiles in primary healthy donor and SLE patient samples. For these studies, we focused on Mo from genotyped healthy donors and SLE patients given our recent findings of elevated IRF5 expression and constitutive activation in these cells [15], [37]. Similar to the molecular cloning described above, a portion of PCR-amplified full-length *IRF5* cDNA from Mo was subjected to deep sequencing analysis. cDNA libraries were generated and sequenced on the ABI SOLiD 3 Plus system. At least 2 million 50-bp reads were obtained from each sample, providing an unprecedented coverage depth of *IRF5*>3,000-fold. To achieve this depth of coverage with traditional molecular cloning, we would have to sequence 27,000 clones per donor. The number of reads obtained per sample are given in Table S1.

**Figure 2:**
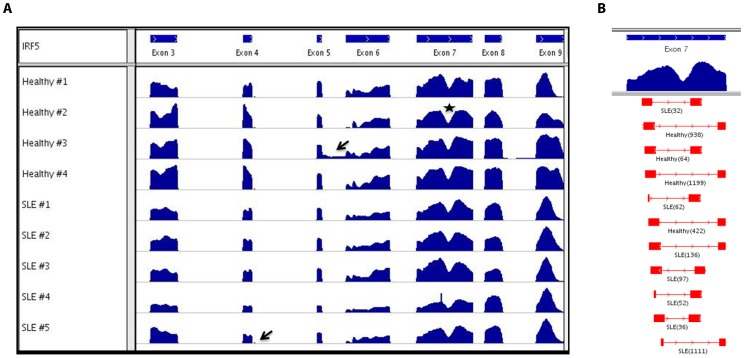
Pile-up view of reads from deep sequencing of PCR-amplified *IRF5* fragments. *A,* Pile-up view of *IRF5* sequencing reads mapped to the *IRF5* genomic sequence was generated with IGV. Peak heights represent the relative frequency of mappings to the indicated regions - hills reflect a high mapping frequency of reads to a particular region and valleys indicate low mapping frequency or deletions (example shown with star); arrows point to areas of reads aligning to previously unannotated exons. Results shown are from 4 independent healthy donors and 5 independent SLE patients. *B,* Reads from healthy donors and SLE patients that mapped to exon 7, shown by the star in *A* above, and reflect true internal deletions of exon 7 are shown. Junction reads are aligned by position and labeled as to whether it was detected in a healthy or SLE sample; the exact number of reads per sample that contained the junction is shown in parentheses.

A visual perspective of the pile-up view of sequencing reads from individual samples mapped to the full-length *IRF5* genomic sequence reveals strong coverage across the entire exons span (Fig. 2A). In order to determine whether the observed structural features, i.e. "hills" and "valleys", reflect alternative splicing events or just position/sequence-bias, we examined reads mapping to the common valley seen in exon 7. We found multiple individual 50 bp reads that contain the exon 7 deletion supporting the existence of extensive alternative splicing in this region (Fig. 2B). In some samples, we also observed the utilization of unannotated exons, shown by the arrows. These data provide further evidence of widespread *IRF5* alternative splicing [15], [19].

**Figure 3:**
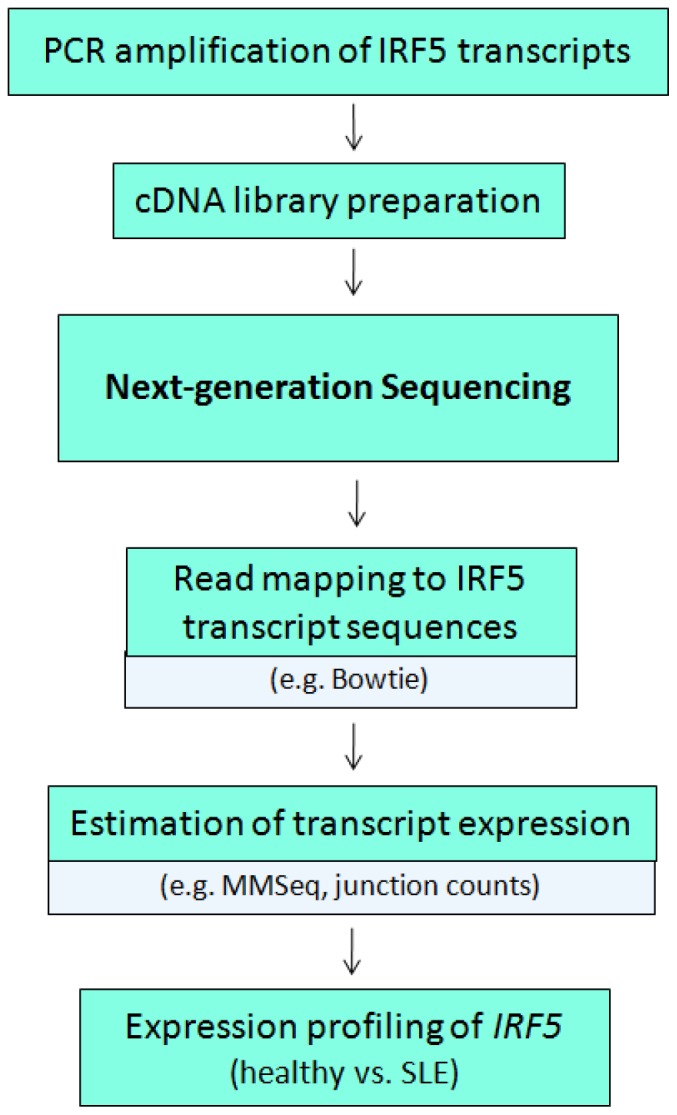
Workflow for enrichment and analysis of single gene profiling by RNA-Seq. Following PCR amplification of RNA of a single gene, cDNA is sequenced by next-generation sequencing and reads are mapped to sequences of alternatively spliced transcripts to obtain expression estimates and to compare transcript expression profiles across samples.

In order to obtain a more accurate and in-depth estimate of the differences between *IRF5* transcript expression in healthy donors and SLE patients, we developed a comprehensive workflow for the analysis of the 50-bp reads obtained from next-generation sequencing (Fig. 3). Estimates of transcript expression were generated in two ways. First, we created an "*IRF5* variant transcriptome" consisting of the 18 transcripts identified from cloning (4 known - V1, V4, V5, V6 and 14 new - NV1-NV14; Fig. 1B). Reads were mapped to this full-length transcriptome and expression profiles between healthy donors and SLE patients were determined. Second, we mapped reads to a list of junctions contained in the *IRF5* variant transcriptome and used junction counts to approximate transcript expression. Results obtained from these two methods are compared.

### Profiling IRF5 Expression by Mapping to the Full-length “IRF5 Variant Transcriptome”

**Figure 4:**
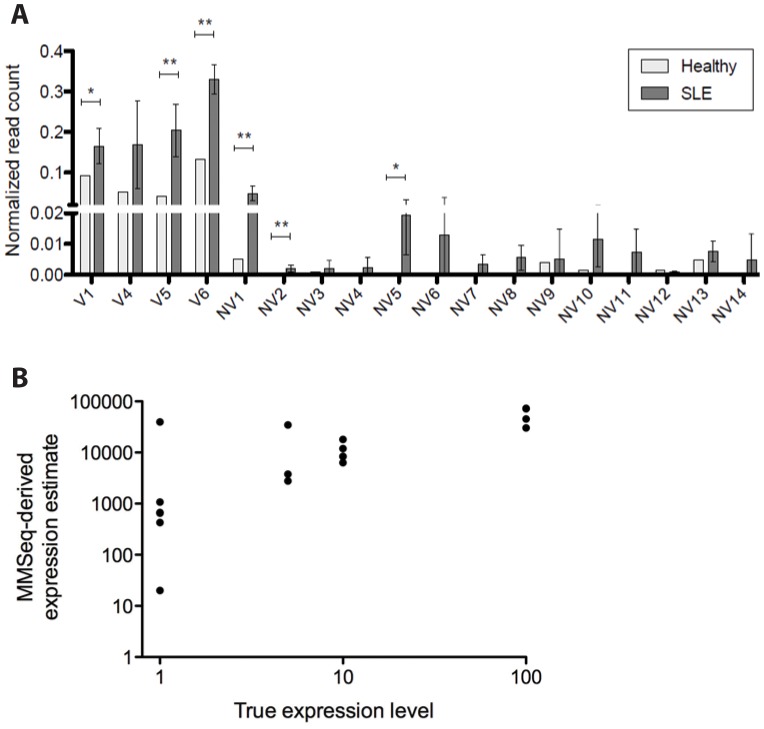
Expression estimates of *IRF5* alternatively spliced transcripts in healthy donors and SLE patients. *A,* Gibbs expression estimates were obtained with MMSeq and converted to raw read counts, then normalized to total read counts. Normalized read counts for each transcript in 5 SLE patients and a representative healthy donor (#1) are plotted; read counts were compared between the two sets using a student's *t*-test. *p<0.05; **p<0.01. *B,* Scatter plot showing the correlation between MMSeq-derived expression estimates and true (assigned) expression levels of the 18 transcript variants. R^2^ = 0.6532 at alpha = 0.05 and *p*<0.0001 indicating a highly significant correlation.

Similar to molecular cloning, next-generation sequencing has its own limitations when it comes to single gene profiling; since each read is only 50 bp in length, it is impossible to obtain complete full-length (∼1600 bp) *IRF5* transcripts from raw sequencing data. As such, in the first approach, we mapped the 50 bp reads from each healthy donor (n = 4) or SLE patient (n = 5) Mo sample to the *IRF5* variant transcriptome and examined their assembly into full-length transcripts in order to obtain expression measures for each transcript. Mapping details are given in the Materials and Methods, as well as Table S1. To estimate the relative abundance of each transcript within a given sample, we used the recently published MMSeq algorithm that accounts for reads that map to multiple transcripts for obtaining expression estimates [39]. Transcript expression between healthy donors and SLE patients was then compared by converting MMSeq estimates (Gibbs values) to count equivalents and normalizing to total mapped reads; this conversion is necessary to compare expression estimates between samples. As a group, SLE patients had significantly higher normalized read counts for some of the alternatively spliced transcripts, providing support for the existence of an *IRF5-*SLE transcript profile that is distinct from that seen in representative healthy donors (Fig. 4A). A comparison of total transcript expression between pooled healthy donors (n = 4) and pooled SLE patients (n = 5) gave a *p* = 0 (10^−10^) by the χ^2^-test supporting a significant difference in *IRF5* transcript profiles between SLE patients and healthy donors. To confirm the validity of this new method for analyzing *IRF5* transcript expression in primary immune cells, we performed a series of simulations to test the MMSeq algorithm, as described in the Materials and Methods. Results from a representative simulation is shown in Fig. 4B supporting the use of MMSeq for estimating the expression of highly similar transcripts. The overall correlation between MMSeq-derived expression estimates and true assigned expression levels of the 18 transcript variants is highly significant with *p*<0.001.

**Figure 5:**
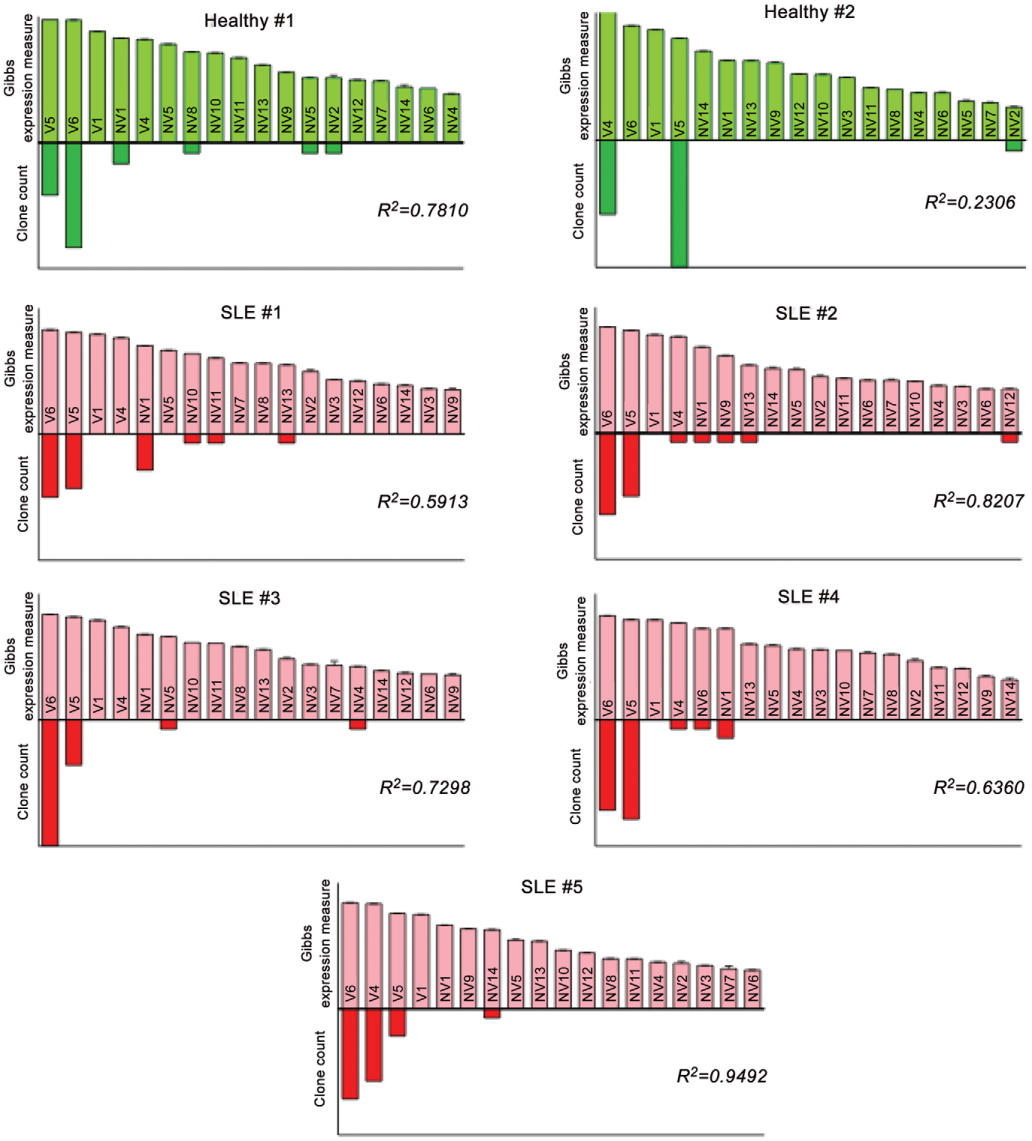
Correlation between Gibbs expression estimates and clone count. Gibbs expression estimates and Monte Carlo standard errors were generated by MMSeq. Bar graphs show Gibbs expression measures from next-generation sequencing versus clone counts from traditional cloning and sequencing. Data from n = 2 healthy donors and n = 5 SLE patients is shown; at least 20 *IRF5*-positive clones from each donor were used for this analysis.

We next compared data obtained from next-generation sequencing to that from molecular cloning to determine whether this new method could be used instead of traditional cloning. By plotting MMSeq model transcript estimates from next-generation sequencing with clone count obtained from molecular cloning, we found that a greater clone count generally correlated with a higher estimated abundance ranking; R^2^ correlation coefficients from scatter plots were all significant (*p*<0.01) at alpha = 0.05 further supporting the validity of this new method (Fig. 5). Data in Fig. 5 from MMSeq analysis are presented as Gibbs expression measures with calculated Monte Carlo standard errors for each transcript. This representation allows us to directly compare, within a single sample, the differential expression between transcripts. A statistical comparison can be made between individual transcripts within a single sample, revealing statistically significant differences between every transcript detected (*p*<0.001). However, the Monte Carlo standard errors are highly simplified in our experimental setup given the depth in our coverage and the redundancy in our transcripts (communicated by Dr. Ernest Turro, author of the MMSeq algorithm [39]); thus, data in Fig. 4A is a “truer” representation of transcript expression allowing for the direct statistical comparison of individual transcripts across samples.

**Figure 6:**
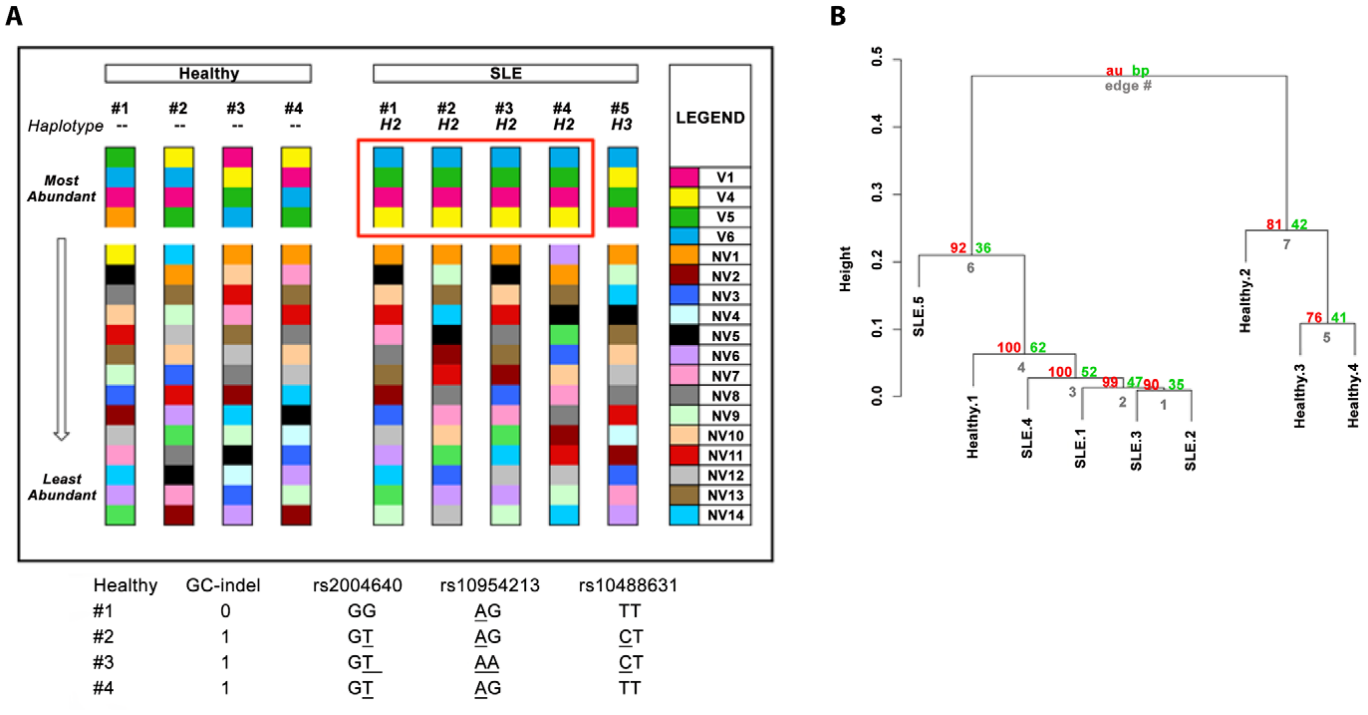
Top four most abundant transcripts are shared by SLE patients with the H2 full risk haplotype. *A,* Relative abundance ranking of transcripts from 4 healthy donors, 1 SLE patient with the H3 full protective haplotype, and 4 SLE patients with the H2 full risk haplotype is shown. Transcripts were ranked from most to least abundant using MMSeq-generated Gibbs expression estimates; individual transcripts are represented by distinct colors. *B,* Hierarchial clustering was performed on the nine samples using expression profiles of the 18 *IRF5* transcript variants. Height represents the difference between samples. Red values are AU *p*-values and green values are BP values. Clusters with AU larger than 95% are statistically significant with a *p*<0.05.

By ranking transcript abundances and stratifying SLE patients by genotype, we found, quite strikingly, that patients with the *IRF5* H2 homozygous full risk haplotype [15] had identical rankings of the top four most abundant transcripts (Fig. 6A). Ranking of these four transcripts in H2 patients was different than in a patient with the H3 homozygous full protective haplotype or healthy donors. Haplotypes of the four healthy donors are shown at the bottom of Fig. 6. Permutation analysis revealed that the probability of observing a shared ranking of the top four transcripts among any four samples by chance is *p* = 2.52×10^−15^, indicating the significance of this abundance profile as being unique to the *IRF5*-SLE H2 risk haplotype. All four H2 samples were estimated to express V6 in the greatest proportion, followed by V5, then V1, then V4. In order to obtain a visual perspective of *IRF5* transcript expression profiles between healthy donors and SLE patients, hierarchial clustering was performed on the nine samples using expression profiles of the 18 *IRF5* transcript variants. Results from this unrestricted analysis provide additional support that the *IRF5* transcript signature found in SLE patients carrying the H2 risk haplotype is highly related to genotype as these four patients (SLE 1–4) clustered very tightly together (Fig. 6B).

### Profiling IRF5 Expression by Junction Counts

**Figure 7:**
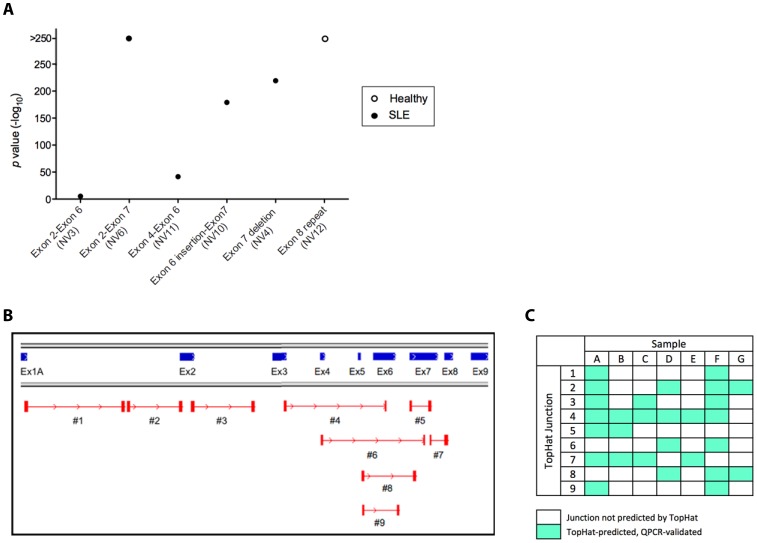
Profiling *IRF5* alternative splicing using junction counts and *de novo* junction discovery. *A,* Counts were obtained for the 6 junctions unique to single cloned transcripts by aligning reads from healthy donor (n = 4) and SLE patient samples (n = 5) to 94-bp junction sequences. Proportionally more transcripts containing each junction were found in either healthy donors (open circles) or SLE patients (closed circles); plotted are the *p* values obtained by the Pearson’s χ^2^-test. *B, De novo* junction discovery was performed using TopHat to identify novel alternative splicing events. Representative junctions are displayed using IGV with the *IRF5* genomic sequence as a reference. Each pair of red blocks represents one junction, and lines joining two blocks denote spliced-out portions of *IRF5*. Junctions 1-3 show evidence of splicing to unannotated intronic regions of *IRF5*; junctions 4–9 have novel inter- and intra-exonic deletions. *C,* Schematic illustration of junctions in *B* confirmed by qPCR.

Another method for comparing *IRF5* transcript expression between healthy donors and SLE patients is by aligning sequencing reads to junctions contained in the *IRF5* variant transcriptome and using counts to approximate transcript expressions. A list of 19 junctions was compiled, each ∼94 bp in length, spanning the joined exons (∼47 bp each of the proximal and distal portions). Junctions are described in Table S2 and sequences listed in Dataset S2. Most of these junctions are shared by multiple variants (Table S3) and thus cannot be used on their own to predict full-length *IRF5* transcript expression. However, we have found that six junctions in the *IRF5* transcriptome are exclusive to single transcript variants (junctions B, C, G, M, Q, and R); thus counts obtained for these 6 junctions might be expected to independently approximate expression of those transcripts. We therefore compared counts for these junctions in pooled healthy donor (n = 4) and SLE patients (n = 5) as a measure of differential expression of these transcript variants between the two groups. Data in Fig. 7A depict that proportionally more transcripts containing junction B (Ex2-6), C (Ex2-7), G (Ex4-6A), M (Ex6insert-7), and Q (Ex7deletion) were found in SLE patients, corresponding to new transcript variants NV3, NV6, NV11, NV10, and NV4, respectively. In healthy donors, we found only transcripts containing junction R (NV12) were elevated. Indeed, we found that these pooled data are in agreement with data from MMSeq analysis of full-length transcript expression (Fig. 4A).

### Expression of IRF5 Transcript Variants Containing the Exon 6 Insertion/deletion

It has recently been demonstrated that a structural insertion/deletion (in/del) variation in exon 6 of the *IRF5* gene is associated with an *IRF5*-SLE haplotype [8], [10]; the presence of the 30-nt insertion (Fig. 1C; exon 6c) is correlated with a risk haplotype for SLE development. This 30-bp in-frame in/del is considered/expected to be a functional genetic variant since it is located in the PEST (proline-, glutamic acid-, serine-, and threonine-rich) domain of IRF5 and protein isoforms that contain alterations in this region, *i.e.* V1, V3/V4, V5 and V2/V6, have differential ability to regulate type I *IFN* promoter reporters [19] and IL6 expression in mouse embryonic fibroblasts (MEFs) [40]. Using the MMSeq model of transcript expression estimates, we were able to demonstrate that as a group, transcripts containing the Ex6c 30-bp deletion (V1, V4, NV7, NV13, NV14) were expressed in significantly lower proportion in SLE patients with the H2 risk haplotype (Table 1). Furthermore, counts for Junction K, which represents the Ex6c deletion, were proportionately lower in these patients as well. As a result, by analyzing our next-generation sequencing data in this way, we were able to detect and support the differential expression of the Ex6C in/del genetic variant in SLE patients carrying the H2 risk haplotype. These data provide independent confirmation of Graham et al's findings [10] and significant support for the screening of the exon 6 in/del in SLE patients as a single pathogenic region conferring risk in SLE.

**Table 1:** Analysis of *IRF5* transcripts containing the Ex6 in/del.

Full-length transcripts	Healthy (Σ read counts)	SLE (Σ read counts)
Containing the Ex6c deletion *(V1, V4, NV7, NV13, NV14)*	7865656^a^	3920591^a^
Missing the Ex6c deletion *(All other variants)*	1054066^a^	2333273^a^
**Junction**	**Healthy** (Σ junction counts)	**SLE** (Σ junction counts)
Exon 6c deletion *(Junction K)*	142709^b^	30640^b^
Other junctions	2606821^b^	574378^b^

Using estimated transcript read counts obtained from MMSeq, expression of transcripts containing vs. those missing the Ex6c deletion was compared in healthy donors (n = 4) and SLE patients carrying the H2 risk haplotype (n = 4). Shown are the total number of reads aligned per group of patients. ^a^χ^2^ = 1377726 with *p*<2.2×10^−16^; *p* values were obtained by the Pearson’s χ^2^-test with Yates continuity correction. Totaled reads mapping to the Ex6C deletion were similarly compared across grouped healthy vs. SLE (risk) patients; ^b^χ^2^ = 16.0379 with *p*<6.209×10^−15^. Σ = sum.

### De Novo Junction Discovery Provides Evidence for Additional IRF5 Transcript Variants

The primary limitation of transcriptome-based expression analysis is that it requires *a priori* knowledge of all the alternatively spliced transcripts, and the use of molecular cloning to generate the transcript sequences may be defined in part by the abundance of a given transcript, *i.e.* we only sequenced ∼20–25 clones per sample and likely missed low-abundant transcripts. Thus, although we observed congruity between expression estimates obtained by quantifying the 18 full-length transcripts in our *IRF5* transcriptome by MMSeq (Figs. 4A) and by quantifying the six junctions that were "unique” to individual transcripts (Fig. 7A), we sought to examine the possibility that additional transcripts exist that are not currently annotated in our transcriptome. Indeed, the raw pile-up view of aligned sample reads (Fig. 2) supports the existence of coding regions within the *IRF5* gene that are not represented in the variants currently contained in our transcriptome. Given these considerations, we hypothesized that additional *IRF5* transcript variants, not yet identified, may be expressed in purified Mo of SLE patients and/or healthy donors. Since next-generation sequencing is unbiased, evidence for the existence of these transcripts should be found in our data; *de novo* junction discovery should help us determine whether additional transcripts exist. We therefore used TopHat software, which compiles exon coverage data, constructs a list of potential splice junctions, and systematically maps the sequencing data to this list to yield a complete set of predicted junctions [41]. We identified a number of new splice junctions that were not found in any of our 18 cloned transcripts, several of which are depicted and described in Fig. 7B and Table 2. In new junctions 1–3, we observed splicing and alignments to areas of *IRF5* which were previously believed to be noncoding, indicating the presence of newly discovered coding exons in *IRF5* full-length transcripts (Fig. 7B). New junctions 4–9 contain deletions of varying sizes, ranging from a novel 218-nucleotide internal deletion in exon 7 (junction 5) to a full deletion of exons 5 and 6 (junction 6).

**Table 2:** Representative novel splice junctions identified by *de novo* junction discovery.

Junction	Predicted spliced regions	Canonical (Yes/No)
1	Ex1a – new exon	Yes
2	New exon – Ex2	Yes
3	Ex2– new exon	Yes
4	Ex3– Ex6	No
5	Ex7– Ex7	No
6	Ex4– Ex7	No
7	Ex7– Ex8	Yes
8	Ex5– Ex7	Yes
9	Ex5– Ex6	Yes

Individual junctions identified by TopHat and illustrated in Fig. 7B are listed and defined as canonical or non-canonical; Ex = exon.

We also analyzed the newly identified junction splice sites to classify as canonical or non-canonical (Table 2) and found that most of the junctions featured the canonical dinucleotides GT and AG, indicating that they reflect a predicted alternative splicing event in *IRF5* and further support that they are not false discoveries generated by random by-products of a large sequencing depth. Further characterization of splice sites identified in our samples by TopHat support that random splicing events are unlikely to contribute significantly to the profile of *IRF5* transcripts detected by the next-generation sequencing analyses in our healthy and SLE patient samples (Figure S1 and Methods S1). Moreover, TopHat also identified the majority of known (canonical and non-canonical) junctions found in our *IRF5* variant transcriptome, thus further supporting the validity of this approach (Table S4).

As a final validation of TopHat and the overall next-generation sequencing approach [42], we used quantitative real-time PCR (qPCR) to examine whether the new junctions reported by TopHat for each healthy donor and patient sample could be detected in the original Mo RNA. Data in Fig. 7C depict the successful confirmation of expression of individual TopHat-discovered junctions within the corresponding samples (Fig. 7C).

### Functional Consequence of an IRF5-SLE H2 Risk Haplotype

**Figure 8:**
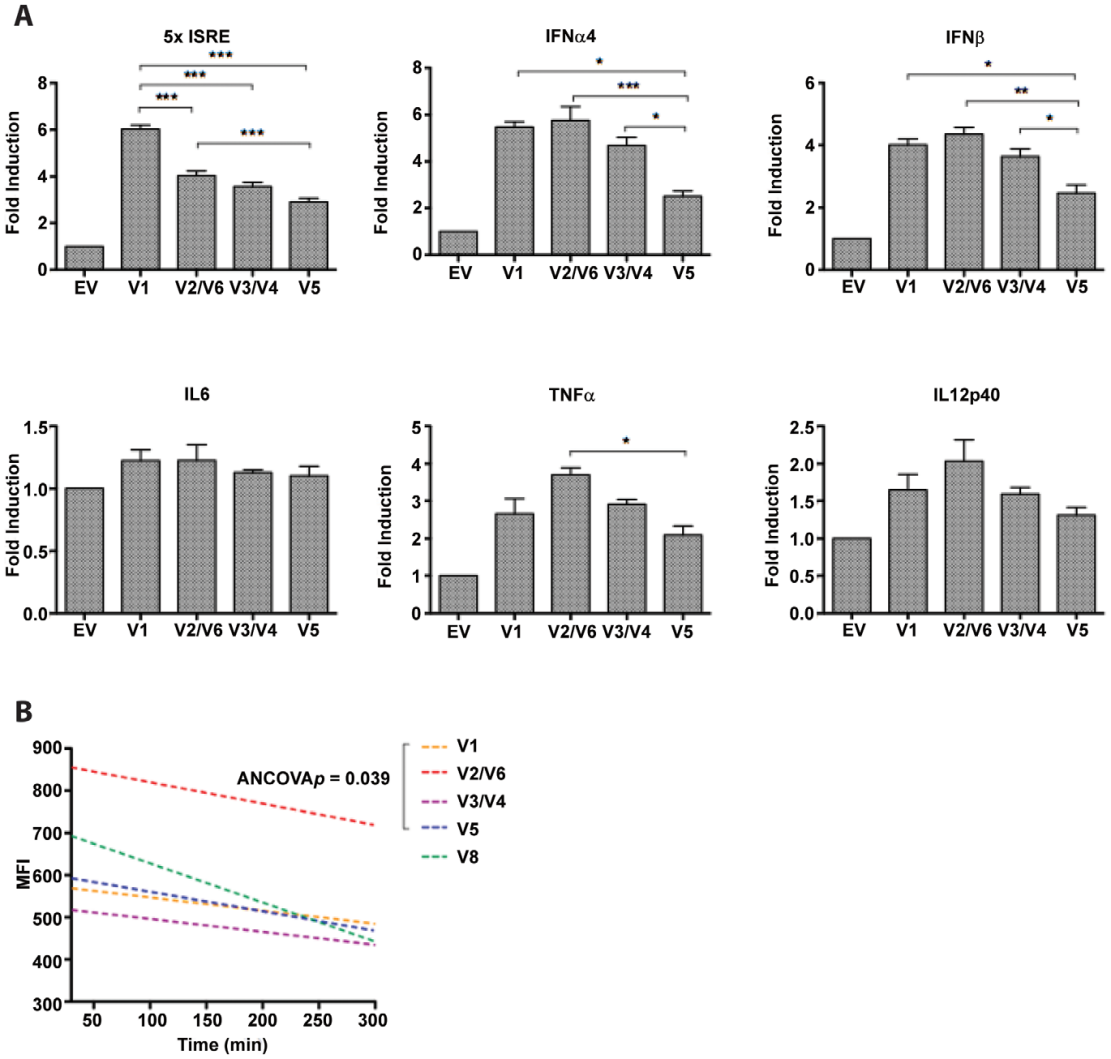
Distinct transactivation potential and stability for IRF5 isoforms associated with the *IRF5*-SLE H2 risk haplotype. *A,* Flag-tagged *IRF5* V1, V2/V6, V3/V4, V5, and empty vector (EV) were co-transfected with the indicated luciferase reporters - 5x*ISRE*, *IFNα4*, *IFNβ*, *IL6*, *TNFα*, or *IL12p40*, and promoter transactivation determined by dual luciferase assay. Independent experiments were repeated in duplicate wells at least three times; mean ± S.D. are plotted. Statistical analysis was performed by one-way ANOVA with Bonferroni post-test; *p<0.05, **p<0.01, ***p<0.001. Promoter induction by each IRF5 isoform was significantly greater than empty vector (EV) controls in all experiments (astericks not shown). *B,* Relative half-lives of IRF5 isoforms V1, V2/V6, V3/V4, and V5 were determined by cycloheximide chase experiments (see Materials & Methods). Similar to *A*, GFP-tagged *IRF5* variants were transfected to Hek293T cells; V8 was included as a comparative control lacking the PEST-rich exons 5–7. Representative data from 2–3 independent experiments is shown as mean fluorescence intensity (MFI) of GFP-IRF5 over the indicated time period. Analysis of covariance (ANCOVA) was used to determine if regression lines between isoforms differed significantly.

Our data thus far supports the existence of an *IRF5* transcript signature in SLE patients that is distinct from that expressed in healthy donors. The dramatic findings that ranking of the top four most abundant transcripts (V1, V4, V5 and V6) and clustering of total transcript signatures are shared by the SLE patients carrying the H2 risk haplotype (Fig. 6) prompted us to investigate further the function(s) of these top four variants that might contribute to SLE risk. Previous studies in human fibroblasts showed that the identical polypeptide encoded by *IRF5* V3/V4 is the most potent inducer of virus-mediated type I IFN [19]. More recent studies indicate that deletions in exon 6 determine the ability of IRF5 isoforms to mediate LPS-induced production of IL6 in MEFs [40]. Given that recent data in *irf5^−/−^* mice reveal a critical role for IRF5 in regulating TLR-mediated proinflammatory cytokines IL6, TNFα, and IL12 [13], which have been shown, along with IFNα, to be pathologically elevated in the circulation of SLE patients [43]–[45], we performed a comparative analysis of human promoter transactivation by the top four most abundant isoforms. Transient transfection to Hek293T cells resulted in equal expression of individual isoforms, as determined by immunoblot (data not shown), yet differential transactivation of each promoter was observed, except for the *IL6* promoter in which all isoforms gave similar transactivation (Fig. 8A). The patterns of transactivation were fairly well-conserved with isoform V2/V6 showing the greatest constitutive transactivation, followed by V1, then V3/V4 and V5. Interestingly, a significantly different pattern of transactivation was observed with the artificially constructed reporter containing 5 copies of the ISRE consensus sequence.

Another manner by which we compared functional differences between these isoforms was by measuring protein stability. For years, others and we have hypothesized [8], [19], [40] that alterations in exon 6 might affect protein stability since this region localizes to the PEST domain [46]–[47]. These top four most abundant isoforms differ only in their exon 6 deletion pattern; V5 has no deletions, V1 has the single 30 bp Ex6c deletion, V2/V6 has the single 48 bp Ex6a deletion, and V3/V4 contains both Ex6a and 6c deletions [19]. Using a modified cycloheximide chase technique [48], we determined the relative stability of GFP-tagged isoforms (Fig. 8B). GFP was used as a positive control with a known 26 hr half-life [49] and V8 as a control isoform lacking the PEST domain [19]. Results from analysis of covariance (ANCOVA) reveal that there is statistical difference between the slopes of the regression lines of these top four most abundant isoforms (Fig. 8B). Multiple comparison/post-hoc testing identified a statistically significant difference between V3/V4 and V5, V3/V4 and V2/V6, V1 and V2/V6, V5 and V2/V6, with *p* values <0.05. The absolute slopes of the regression lines for each isoform indicated that the most stable was V3/V4≅V1<V5<V2/V6<V8, with V8 being the least stable. As expected, GFP gave the greatest relative stability, with only a small decrease in mean fluorescence intensity (MFI) over time (data not shown). Surprisingly, based on the PEST domain alone, we would have predicted a different pattern of stability, with V8 being the most stable and V5 being the least. These data indicate that the PEST domain of IRF5 is not the determining factor for relative stability.

## Discussion

High-throughput mRNA sequencing (RNA-Seq) of mammalian transcriptomes can provide a wealth of information on transcript assembly and abundance estimates [50]. Major challenges arise, however, when attempting to measure the differential expression of multiple alternatively spliced transcripts from a single gene [51]–[53]. Given that many of these transcripts will be expressed at low levels [54], we chose to enrich for our gene of interest first, before sequencing, in order to alleviate some of these later challenges in analysis. While enrichment of *IRF5* by PCR amplification may introduce artifactual changes in transcript quantity due to structural variation in the material being sequenced; for example, certain nucleotide strings may be less efficiently amplified than others or they may form higher-order structures that cannot be amplified, we would expect this to be minimized in the current study given the high similarity and redundancy in *IRF5* transcript variants. *IRF5* alternatively spliced transcripts are a family of very similar molecules, thus the efficiency of PCR amplification should theoretically be similar for all transcripts.

To further enhance our ability to analyze results from next-generation sequencing, we used data from molecular cloning to generate an *IRF5* variant transcriptome. We developed a newly defined workflow of next-generation sequencing whereby differentially spliced transcripts of a single gene could be rapidly enriched and quantified in donor RNA samples. Results obtained from these various steps of the pipeline confirmed what was found by molecular cloning but at a much greater depth. Some examples of this include, but are not limited to, the finding that 1) MMSeq estimates were comparable to cloning estimates (Fig. 5), and 2) TopHat *de novo* discovery detected essentially all of the junctions found in our newly cloned transcripts (Table S4). Using these methods, we were also able to confirm previous findings that support a role for the Ex6c in/del in SLE (Table 1) [8], [10]. Altogether, findings presented herein support the use of this newly defined workflow of next-generation sequencing to 1) confirm the contribution of genotype to the profile of *IRF5* transcripts expressed, and 2) address the question of what *cis* polymorphisms in the *IRF5*-SLE haplotype regulate *IRF5* alternative splicing? Both of these studies will require sequencing on larger cohorts of genotyped healthy donor and SLE patient samples; however, the first critical support and proof-of-principle for additional studies, such as these, is now provided. Data in the current study support a significant role for genotype in defining the top four most abundant transcripts expressed, yet little is still known of the mechanisms regulating *IRF5* alternative splicing. *Cis*-acting variants have been identified in the *IRF5* gene that associate with elevated expression, but the data on *cis* regulation of *IRF5* alternative splicing is unclear [7]–[8], [55]. Indeed, recent findings by Alonso-Perez et al. indicate that *cis*-regulation of *IRF5* expression is not enough to fully account for *IRF5* association with SLE susceptibility, suggesting that other mechanisms exist that regulate functional changes in *IRF5* [56]. These data are confirmatory of earlier work in the lab revealing that genotype alone was not enough to account for elevated IRF5 expression in primary immune cells of SLE patients and enhanced *IRF5* alternative splicing was, at least in part, due to elevated levels of spliceosome components in SLE patients as overexpression of many of these factors led to increased *IRF5* alternative splicing [15].

There are currently numerous software programs available for the assembly and analysis of the short reads obtained by RNA-Seq. Due to the high level of redundancy in our transcriptome (Fig 1B), after testing a number of these programs (MMSeq, Cufflinks, RSEM), we chose to primarily use MMSeq to quantify *IRF5* transcript expression since its algorithm was specially designed to account for reads that map to multiple transcripts [39]. We found that many of the other expression estimate algorithms discard these reads, thus underestimating expression [39]. Indeed, simulation experiments testing the validity of this approach, whereby arbitrary expression values were assigned to each of the 18 transcripts and reads simulated using WGSIM [57], gave significant correlation between the assigned true expression level and MMSeq-derived expression estimates (Fig. 4B). We found in the simulations that MMSeq is most accurate with the more abundant transcripts thus providing additional confidence in the statistical findings of Fig. 4A since significance was only obtained in the more abundant transcripts. This makes sense since the relative error in MMSeq estimates is larger for less abundant transcripts and relatively small for highly abundant transcripts based on the algorithm used by MMSeq that assigns reads to each iteration probabilistically [39].

Our results also support the use of next-generation sequencing to broaden the *IRF5* transcriptome contained in primary immune cells of SLE patients and healthy donors. The unbiased junction discovery performed by TopHat produced an assortment of new junctions whose expression was confirmed by qPCR in original RNA from healthy donors and SLE patients (Fig. 7B&C). Ultimately, these data suggest that new full-length transcripts could be cloned and sequences input back into the pipeline (Fig. 3) so as to obtain an even closer approximation of the "true" *IRF5* transcript signature in SLE patients. Moreover, the successful *in vitro* detection of these new junctions derived solely from raw next-generation sequencing reads provides additional validation for the use of RNA-Seq in *IRF5* transcript expression profiling.

Results from these studies significantly enhance our current understanding of *IRF5* expression in monocytes of SLE patients and provide critical insight into how genotype might contribute to and/or alter IRF5 function. Further studies will be required to determine if unique transcript profiles will be obtained between purified B cells of SLE patients, where IRF5 has recently been proposed to function [32], [58], and Mo, and whether a distinct difference in transcript expression will be necessary to predict altered function(s) for IRF5 in these two immune cell populations. Data in Table 1, from analyzing the exon 6 in/del, supports that this region alone may be sufficient to define SLE risk and thus could be used as a potential marker of IRF5 pathogenic function. Future studies will be important to confirm this finding in additional healthy donor and SLE patient samples, along with determining the functional consequence of the in/del as many variations in exon 6 have been observed (Fig. 1B).

Conversely, it may be that all immune cells share the same *IRF5* pathogenic signature in SLE. Nevertheless, data from all three methods of analyses (molecular cloning, MMSeq, and junction counts) support the presence of an *IRF5*-SLE transcript signature in purified Mo of SLE patients that is significantly different from that expressed in healthy donor Mo. To this end, we provide intriguing data to be built upon in the near future that suggests genotype may define, not only the top four most abundant transcripts expressed in Mo of SLE patients with the H2 risk haplotype, but the *IRF5* transcript signature itself, as patients carrying the H2 risk haplotype clustered tightly together (Fig. 6B). It is important to point out the relevance of the different abundance rankings as slight changes in the order of the top four most abundant transcripts are likely to result in significant differences in expression. This is best shown in Fig. 4A through the statistical comparison of normalized expression levels between healthy donor #1, that has the abundance ranking of **V5**> **V6**> V1> NV1, and patients with the H2 haplotype (**V6**> **V5**> V1> V4). We found a significant difference in the levels of V5 and V6 between the healthy donor and SLE patients even though these top two ranked variants only changed order position. Whether all of these data together define the exact *IRF5*-SLE pathogenic signature that delineates its global function in SLE is still unclear. Future studies will be necessary that focus on identifying new transcripts, determining their abundance, and characterizing their function(s) in the immune system.

To this extent, we present data that supports the premise that IRF5 isoforms have differential abilities to regulate proinflammatory cytokines associated with SLE pathogenesis (Fig. 8) [19], [40]. In this study, we focused on characterizing further the function of the top four expressed transcripts (V6>V5>V1>V4) identified in SLE patients with the H2 risk haplotype. We found that V6 had the greatest transactivation function yet the lowest stability of the four isoforms (V4>V1>V5>V6). These data are intriguing and support a complex scenario in SLE where a particular IRF5 isoform might have enhanced transactivation potential, yet decreased relative stability that may instead diminish its pathologic contribution. An important question to be addressed in future studies is - what is the functional consequence of an altered *IRF5* expression profile? Current preliminary data on some of the new variants has yet to point toward distinct functions (data not shown) [19]; although, one might argue the relevance of analyzing the function of individual encoded isoforms since it is clear in SLE that isoforms are not expressed in a singular fashion and instead are expressed all together. IRF5, like other IRF family members, forms homodimers and/or heterodimers with other proteins resulting in function [26], [59]–[60]. The complexity of this system is thus obvious and future studies on isoform function will have to take into account the aspects of differential protein stability, DNA binding potential and interaction with other IRF5 isoforms and proteins. Additional work in this area will be essential to our understanding of exactly how elevated and altered *IRF5* transcript expression in SLE patients contributes functionally to disease pathogenesis.

## Materials and Methods

### Ethics Statement

All study subjects provided written informed consent to participate in the study, and the study was approved by institutional and regional ethical boards at both the University of Medicine and Dentistry of New Jersey and Uppsala University.

### Study Participants and Genotyping

Purified monocyte (Mo) samples from 6 Swedish patients with SLE were obtained from the rheumatology clinic at Uppsala University Hospital (UUH) in Sweden; Mo (n = 4) from healthy controls were obtained from Uppsala University Hospital. Each of the patients fulfilled at least four of the classification criteria for SLE as defined by the American College of Rheumatology (ACR) with a median ACR index of 6 (range 5–8). Clinical disease activity at the time of blood sampling was assessed with the modified SLE disease activity index (mSLEDAI-2K) where complement and anti-DNA antibodies were omitted. The median mSLEDAI-2K value was 1 (range 0–26) showing that most patients had low or no clinical disease activity at the time of blood collection.

### Genotyping

Subjects were genotyped at the rs2004640, rs10954213, and rs10488631 SNPs in 250 ng of DNA extracted from blood samples of study subjects using the Illumina Golden Gate assay (Illumina, San Diego, CA, USA), as described [4], [16]. The 5 bp CGGGG indel of *IRF5* was genotyped by PCR amplification followed by size separation using 4% agarose gels, or using an ABI 3770 capillary sequencer (Applied Biosystems, Foster City, CA, USA).

### RNA Extraction and PCR Amplification

Mo were purified immediately following phlebotomy from peripheral blood of healthy donors and SLE patients by positive selection with CD14 magnetic beads (Miltenyi Biotech). RNA was extracted using the RNEasy Mini kit (Qiagen) and genomic DNA was removed by DNase treatment (Promega). One-step RT-PCR Kit (Qiagen) was used to amplify full-length *IRF5* transcripts from non-coding Ex1A through 9. PCR conditions were as follows: 1 cycle of 50°C for 30 minutes and 95°C for 15 minutes, followed by 30–40 cycles of 94°C for 30 seconds, 62°C for 30 seconds, and 68°C for 2 min 30 seconds, then 1 cycle of 72°C for 10 minutes. In some cases, a second round of nested PCR was performed with an exon 2 primer spanning the ATG start codon sequence. PCR products were separated by agarose gel electrophoresis on a 1% gel. The amplified band corresponding to MW 1600 bp was excised and purified with the QiaQuick gel extraction kit (Qiagen) and used for downstream cloning/sequencing.

### Molecular Cloning of Transcripts

Gel-purified PCR products were ligated into the pCR®2.1-TOPO vector (Invitrogen) or pDrive UA cloning vector (Qiagen) and heat-transformed as per manufacturer’s instructions. Following blue/white selection, plasmids were extracted from individually grown colonies with the Plasmid Miniprep kit (Qiagen) and checked for correctly sized inserts by EcoR1/BamH1 digestion. Plasmids were sequenced with the ABI 3130xl Genetic Analyzer (Applied Biosystems, Inc., Foster City, CA) using M13R and M13F sequencing primers. Forward and reverse sequences were assembled and analyzed using CLC DNA Workbench 5 software (CLC bio, Cambridge, MA). Annotations were made to indicate exons and junctions (known or novel) present in each transcript. A minimum of 20 *IRF5*-positive clones were sequenced and identified from each individual donor. Sequences of novel variants NV1-NV14 are found in Dataset S1.

### High-throughput Sequencing

PCR products were sheared into 100–150 base pair (bp) fragments, ligated to sequencing adapters, nick-translated, and amplified to yield a library for each individual. Gel-purified PCR products from each donor were sheared into 100–150 bp fragments and ligated to barcoded sequencing adapters. The resulting products were nick translated and amplified three to five cycles to yield a library for each donor. These libraries were attached to beads by emulsion PCR and sequenced by ligation on the ABI SOLiD 3 plus system (Life Technologies, Carlsbad, CA). Greater than 2×10^6^ 50-bp reads were obtained from each sample.

### Read Mapping

Bowtie software [61]–[62] was used to perform all alignments, using default mapping/quality parameters unless otherwise specified below. Mapping statistics for each set of alignments are found in Table S1. For MMSeq-based analyses, reads were aligned to an index of the 18 *IRF5* transcript sequences identified by cloning. Parameters were set to report all alignments of equivalent alignment score, thus accounting for reads that could originate from multiple transcripts sharing common exons (Bowtie parameters “–a –best –strata”). SAMTools software [57] was used to convert alignment files to BAM format. Mappings of reads to the transcript set were generated using MMSeq feature “bam2hits” in preparation for running the MMSeq software pipeline [39]. For junction-based quantitation, reads were aligned to an index of 19 junction sequences (94 bp each) obtained from sequences of cloned transcripts (Table S2). Bowtie parameters were set to report the single best alignment for each read (“-k 1–best”). For alignments to the *IRF5* gene, an index was built from the *IRF5* genomic sequence retrieved from NCBI RefSeq# NC_000007.13 (128577994.128590089) on Chromosome 7. Reads were mapped with parameters “-n 2–k 40–m 40.”

### Determination of Coverage Depth and Cloning Estimates

A minimum of 800,000 50-bp reads per sample aligned to *IRF5* sequences (Table S1). The *IRF5* gene is approximately 12,000 bp; 800,000 reads×50 bp per read/12,000 bp in *IRF5* =  a minimum of 3,000-fold coverage of *IRF5.* Each *IRF5* alternatively spliced transcript is approximately 1600 bp, which corresponds to ∼30 50-bp sequencing reads. 800,000 reads divided by 30 reads per transcript yields the equivalent of 27,000 *IRF5* transcript-containing clones.

### Transcript Expression Estimates and Abundance Rankings with MMSeq

Following mapping of reads to the *IRF5* transcriptome, alignments were input to the MMSeq pipeline [39] to obtain mean log Gibbs expression estimates and Monte Carlo standard errors for each of the 18 transcripts within a given donor sample. Abundance rankings were obtained by listing transcripts in order of magnitude of Gibbs values within each sample. To obtain “counts” for comparing transcript expression across samples, Gibbs expression measures for each transcript were converted to raw read counts by multiplying by the transcript length (in kb) and then by the total number of reads (in millions) in the alignments file from each sample. Raw read counts were then normalized to total read counts in the sample, and values were compared across healthy and SLE samples.

### Transcript Expression Estimates by Quantification of Unique Junctions

Counts for each of the junctions unique to one of the 18 alternatively spliced transcripts were used to construct six 2×2 contingency tables. Rows contained pooled counts of each unique junction vs. counts of other junctions (rows) for grouped healthy donors vs. SLE patients (columns). The Pearson’s χ^2^-test was then performed on each 2×2 contingency table; the two-tailed *p*-value reported represents the probability of observing the data by chance if there is no differential expression between healthy donors and SLE patients.

### MMSeq Simulations

Arbitrary expression values of 1, 5, 10, or 100 were randomly assigned to each of the 18 transcript variants. 50 bp reads were simulated separately for each transcript by WGSIM, part of the SAMtools distribution [57], at high coverage (>600x) so as to minimize sampling bias. Read mapping and MMSeq-based analyses was performed as described above.

### Analysis of Exon 6C Insertion/deletion

The χ^2^-test was used to examine the differential expression of the exon 6C in/del in healthy donors vs. SLE patients. Raw read counts from MMSeq-based estimates were summed to obtain totals for transcripts containing vs. not containing the exon 6C deletion; counts for multiple transcripts in each group of healthy vs. SLE patient samples formed the rows of a contingency table. Alignments to Junction K (representing the Exon 6C deletion) vs. other junctions were similarly totaled for both groups. The Pearson’s χ^2^-test was performed on each 2×2 contingency table; the two-tailed *p*-value reported represents the probability of observing the data in the contingency table by chance if there is no differential expression between healthy donors and SLE patients.

### De Novo Identification and Confirmation of Splice Junctions

Using Bowtie default mapping parameters contained within the TopHat software pipeline [41], reads were aligned to the *IRF5* genomic sequence (NCBI RefSeq# NC_000007.13 (128577994.128590089)), and iterative mappings to predicted splice junctions were performed and quantified by TopHat. To control for false positive junctions found by TopHat, splice sites of reported junctions were classified as canonical or non-canonical; a junction was considered non-canonical if its splice site was none of the following: GT…AG, GC…AG, AT…AC, GG…AG, GT…TG, GT…GG, CT…AG [63]–[64]. A threshold value of 0.01% of total mapped reads, where the fraction of non-canonical junction sites levels off, was set to exclude junctions reported by chance (described further in Method S1 and Figure S1).

To confirm TopHat findings, junction-specific quantitative PCR (qPCR) was performed on original monocyte RNA using a modified approach described in [42]. Individual primer sets (shown in Table S5) were designed with one primer corresponding to the splice-specific region of each junction (containing portions of the two spanned exons), and the other primer corresponding to neighboring exonic sequences; detection of an amplified product confirmed junction presence. qPCR conditions were: 1 cycle of 45°C for 3 minutes and 95°C for 10 minutes, followed by 40 cycles of 95°C for 15 seconds and 60°C for 1 minute.

### Dual Luciferase Assays and Protein Stability

HEK-293T cells were transfected using Lipofectamine 2000 (Invitrogen). For dual luciferase assays, a 1∶1 ratio (2 µg each) of luciferase reporter and IRF5 expression plasmid along with 0.1 µg of pRL-CMV as an internal control were used. Cells were harvested 24 h post-transfection and lysed with Passive Lysis Buffer (Promega). Luciferase assays were carried out using the Dual Luciferase Assay kit according to the manufacturer’s specifications. Levels of reporter firefly luciferase activity were normalized to a constant level of pRL-CMV activity; values were normalized to the luciferase readings from an empty vector control. Experiments were repeated at least three times in duplicate and data are presented as fold induction; mean ± S.D. are plotted. Statistical analysis was performed by one-way ANOVA with Bonferroni post-test. For the determination of IRF5 stability, cells were transfected with 8 µg GFP-IRF5 plasmids. 48 hr post-transfection, cells were fractionated by fluorescence activated cell sorting (FACS) into subpopulations expressing low, medium, and high levels of GFP, as described [48]. Cells with medium expression of GFP were treated with 100 µg/mL cycloheximide for 5 hr. Cells were collected at 30–60 min intervals and analyzed by flow cytometry for mean GFP fluorescence. Linear regression analysis of mean fluorescence vs. time was performed for each isoform, and analysis of covariance (ANCOVA) with multiple comparison test (by Tukey's HSD test and Bonferroni's post-hoc) was used to determine if regression lines between isoforms differed significantly.

### Data Display

The Integrative Genomics Viewer (IGV) [65] was used to display transcripts, read alignments, and junctions as they map to coordinates of the *IRF5* genomic sequence.

### Statistical Analysis of IRF5 Expression Profiles in Healthy vs. SLE

The two-way ANOVA with Bonferroni post-test was used to detect differences in *IRF5* transcript profiles obtained by cloning from healthy and SLE Mo. For the testing of differential expression between healthy donors and SLE patients, the mean ± S.D. of normalized raw read counts (MMSeq-based predictions) or normalized expression level for each transcript was calculated and pooled for the SLE subjects, and the student's *t*-test was used to test the null hypothesis that a healthy subject is the same as the SLE group. Pearson correlation coefficient was used to detect differences between MMSeq-derived expression estimates and true expression levels (in the simulation study) and MMSeq-derived Gibbs expression measures and clone count at alpha = 0.05 with a 95% confidence interval. Hierarchial clustering on the samples using expression profiles of the 18 transcripts was performed by multiscale bootstrapping using the pvclust package of R.

### Calculating the Probability of Transcript Rankings

Given 7 independent subjects, where each subject has 18 transcripts, we used combinatorics to determine the probability that the top 4 most abundant transcripts in any 4 subjects are the same: *p* = (14!/18!)^3^((18!-4!14!)/18!)^3^ = 2.522181E^−15^.

## Supporting Information

Figure S1 **Filtering TopHat junctions by relative hits excludes non-canonical junctions likely to represent random splicing.** TopHat-reported junctions for two representative samples (1 healthy donor, 1 SLE patients) are grouped by “relative hits”, or the percentage of total mapped reads aligning to them, and the fraction of junctions containing a non-canonical splice site is plotted versus the relative hits %. The frequency of non-canonical junctions levels off as the relative hits % approaches 0.01%.
(TIF)

Table S1 **Read mapping statistics.** For each sequenced healthy donor and SLE patient sample, the number of reads as well as percentages of reads mapped within each Bowtie alignment is shown. For MMSeq-based expression estimates, mapping was performed to transcript variants (column 3); to *IRF5* genomic sequence for pile-up views (column 4); to junction sequences for junction-based expression estimates (column 5); and iteratively to *IRF5* and its predicted junctions by TopHat *de novo* junction detection software (column 6).
(TIF)

Table S2 **List of splice junctions identified in the** ***IRF5*** **variant transcriptome.** Junctions were considered canonical if splice sites consisted of nucleotides GT…AG, GC…AG, AT…AC, GG…AG, GT…TG, GT…GG, or CT…AG [62]. Del = deletion; ins = insertion; N/A - not applicable (*junction R represents an internal duplication).
(TIF)

Table S3 **List of junctions contained in each full-length alternatively spliced** ***IRF5*** **transcript.** Each of the 18 transcripts contains a unique combination of junctions.
(TIF)

Table S4 **List of junctions in the** ***IRF5*** **variant transcriptome that were detected by TopHat.** Junctions were considered canonical if splice sites consisted of nucleotides GT…AG, GC…AG, AT…AC, GG…AG, GT…TG, GT…GG, or CT…AG [62]. Individual junctions present in each sample are shown by the x.
(TIF)

Table S5 **List of primers used to confirm TopHat discovered junctions 1–9.**
(TIF)

Dataset S1 **Sequences of the 14 novel alternatively spliced** ***IRF5*** **transcripts.** New variants were identified by cloning from purified monocytes of healthy donors and SLE patients.
(DOC)

Dataset S2 **Sequences of the 19 junctions derived from the** ***IRF5*** **variant transcriptome.** Each junction is 94 bp in length and consists of 47-bp blocks of the two spliced exons at the area of intersection.
(DOCX)

Method S1 **Filtering** ***de novo*** **junctions reported by TopHat to exclude random splicing.** True splice junctions are more likely to have a canonical or near canonical splice site, while random (non-significant) splicing is more likely to use non-canonical splice sites [62]–[63]; thus the fraction of non-canonical sites among junctions reported can serve as an indicator of the false positive rate. Data in Table S2 reveal that the majority of junction splice sites found in the *IRF5* variant transcriptome feature the canonical dinucleotides GT and AG as intronic donor and acceptor splice sites, respectively. However, well-known *IRF5* variants V1 and V4 contain the non-canonical junction K that generates one of the Ex6 deletions [19]. Thus, validity of junctions discovered *de novo* might better be determined with a cut-off. When we define “relative hits” as the number of reads mapping to a TopHat-reported junction per fraction of all reads for all reported junctions, we observe that the fraction of non-canonical splice sites for discovered junctions decreases when the number of relative hits to the junctions increase (Figure S1). When the relative hits for a junction reaches 0.01%, the frequency of having a non-canonical splicing site tends to level off to near zero. Based on these findings, we set a threshold of 0.01% and discard reported junctions with relative hits below this value, thus more likely including junctions that represent true, non-random splicing.
(TIF)

## References

[1] Rahman A, Isenberg DA (2008) Systemic lupus erythematosus. N Engl J Med 358: 929–939. 18305268 10.1056/NEJMra071297

[2] Flesher DL, Sun X, Behrens TW, Graham RR, Criswell LA (2010) Recent advances in the genetics of systemic lupus erythematosus. Expert Rev Clin Immunol 6: 461–479. 20441431 10.1586/eci.10.8 PMC2897739

[3] Deng Y, Tsao BP (2010) Genetic susceptibility to systemic lupus erythematosus in the genomic era. Nat Rev Rheumatol 6: 683–692. 21060334 10.1038/nrrheum.2010.176 PMC3135416

[4] Sigurdsson S, Nordmark G, Harald Goring HH, Lindroos K, Wiman C, et al (2005) Polymorphisms in the tyrosine kinase 2 and interferon regulatory factor 5 genes are associated with systemic lupus erythematosus. Am J Hum Genet 76: 528–537. 15657875 10.1086/428480 PMC1196404

[5] Demirci FY, Manzi S, Ramsey-Goldman R, Minster RL, Kenney M, et al (2006) Association of a common interferon regulatory factor 5 (IRF5) variant with increased risk of systemic lupus erythematosus (SLE). Ann Hum Genet 71: 308–311. 17166181 10.1111/j.1469-1809.2006.00336.x

[6] Cunninghame Graham DS, Manku H, Wagner S, Reid J, Timms K, et al (2007) Association of IRF5 in UK SLE families identifies a variant involved in polyadenylation. Hum Mol Genet 16: 579–591. 17189288 10.1093/hmg/ddl469 PMC3706933

[7] Graham RR, Kozyrev SV, Baechler EC, Reddy MV, Plenge RM, et al (2006) A common haplotype of interferon regulatory factor 5 (IRF5) regulates splicing and expression and is associated with increased risk of systemic lupus erythematosus. Nat Genet 38: 550–555. 16642019 10.1038/ng1782

[8] Kozyrev SV, Lewen S, Reddy PM, Pons-Estel B (2007) Argentine Collarborative Group, et al (2007) Structural insertion/deletion variation in IRF5 is associated with a risk haplotype and defines the precise IRF5 isoforms expressed in systemic lupus erythematosus. Arthritis Rheum 56: 1234–1241. 17393452 10.1002/art.22497

[9] Shin HD, Sung YK, Choi CB, Lee SO, Lee HW, et al.. (2007) Replication of genetic effects of interferon regulatory factor 5 (IRF5) on systemic lupus erythematosus in a Korean population. Arthritis Res Ther 9, R32. 10.1186/ar2152 PMC1906810 17389033

[10] Graham RR, Kyogoku C, Sigurdsson S, Vlasova IA, Davies LR, et al (2007) Three functional variants of IFN regulatory factor 5 (IRF5) define risk and protective haplotypes for human lupus. Proc Natl Acad Sci USA 104: 6758–6763. 17412832 10.1073/pnas.0701266104 PMC1847749

[11] Barnes BJ, Moore PA, Pitha PM (2001) Virus-specific activation of a novel interferon regulatory factor, IRF-5, results in the induction of distinct interferon alpha genes J Biol Chem. 276: 23382–23390. 10.1074/jbc.M101216200 11303025

[12] Schoenemeyer A, Barnes BJ, Mancl ME, Latz E, Goutagny N, et al (2005) The Interferon Regulatory Factor, IRF-5, Is a Central Mediator of Toll-like Receptor 7 Signaling. J Biol Chem 280: 17005–17012. 15695821 10.1074/jbc.M412584200

[13] Takaoka A, Yanai H, Kondo S, Duncan G, Negishi H, et al (2005) Integral role of IRF-5 in the gene induction programme activated by Toll-Like Receptors. Nature 434: 243–249. 15665823 10.1038/nature03308

[14] Yanai H, Chen HM, Inuzuka T, Kondo S, Mak TW, et al (2007) Role of IFN regulatory factor 5 transcription factor in antiviral immunity and tumor suppression. Proc Natl Acad Sci USA 104: 3402–3407. 17360658 10.1073/pnas.0611559104 PMC1805533

[15] Feng D, Stone RC, Eloranta ML, Sangster-Guity N, Nordmark G, et al (2010) Genetic variants and disease-associated factors contribute to enhanced IRF-5 expression in blood cells of systemic lupus erythematosus patients. Arthritis Rheum 62: 562–573. 20112383 10.1002/art.27223 PMC3213692

[16] Sigurdsson S, Goring HHH, Kristjandsdottir G, Milani L, Nordmark G, et al (2008) Comprehensive evaluation of the genetic variants of interferon regulatory factor 5 reveals a novel 5 bp length polymorphism as strong risk factor for systemic lupus erythematosus. Hum Mol Genet 17: 872–881. 18063667 10.1093/hmg/ddm359

[17] Niewold TB, Hua J, Lehman TJ, Harley JB, Crow MK (2007) High serum IFN-alpha activity is a heritable risk factor for systemic lupus erythematosus. Genes Immun 56: 3995–4004. 10.1038/sj.gene.6364408 PMC2702174 17581626

[18] Niewold TB, Kelly JA, Flesch MH, Espinoza LR, Harley JB, et al (2008) Association of the IRF5 risk haplotype with high serum interferon-α activity in systemic lupus erythematosus patients. Arthritis Rheum 58: 2481–2487. 18668568 10.1002/art.23613 PMC2621107

[19] Mancl ME, Hu G, Sangster-Guity N, Olshalsky SL, Hoops K, et al (2005) Two distinct promoters regulate the alternative-spliced human Interferon regulatory factor-5 variants: Multiple variants with distinct cell-type specific expression, localization, regulation and function. J Biol Chem 280: 21078–90. 15805103 10.1074/jbc.M500543200

[20] Barnes BJ, Kellum MJ, Field AE, Pitha PM (2002) Multiple regulatory domains of IRF-5 control activation, cellular localization and induction of chemokines that mediate T-lymphocyte recruitment. Mol Cell Biol 22: 5721–5740. 12138184 10.1128/MCB.22.16.5721-5740.2002 PMC133975

[21] Barnes BJ, Richards J, Mancl M, Hanash S, Beretta L, et al (2004) Global and distinct targets of IRF-5 and IRF-7 during innate response to viral infection. J Biol Chem 279: 45194–45207. 15308637 10.1074/jbc.M400726200

[22] Barnes BJ, Kellum MJ, Pinder KE, Frisancho JA, Pitha PM (2003) Interferon regulatory factor 5, a novel mediator of cell cycle arrest and cell death. Cancer Res 63: 6424–6431. 14559832

[23] Hu G, Mancl M, Barnes BJ (2005) Signaling through IFN regulatory factor-5 sensitizes p53-deficient tumors to DNA damage-induced apoptosis and cell death. Cancer Res 65: 7403–7412. 16103093 10.1158/0008-5472.CAN-05-0583

[24] Couzinet A, Tamura K, Chen HM, Nishimura K, Wang Z, et al (2008) A cell-type-specific requirement for IFN regulatory factor 5 (IRF5) in Fas-induced apoptosis. Proc Natl Acad Sci U S A 105: 2556–2561. 18268344 10.1073/pnas.0712295105 PMC2268175

[25] Hu G, Barnes BJ (2009) IRF-5 is a mediator of the death receptor-induced apoptotic signaling pathway. J Biol Chem 284: 2767–2777. 19028697 10.1074/jbc.M804744200

[26] Feng D, Sangster-Guity N, Stone R, Korczeniewska J, Mancl ME, et al (2010) Differential requirement of histone acetylase and deacetylase activities for IRF5-mediated proinflammatory cytokine expression. J Immunol 185: 6003–6012. 20935208 10.4049/jimmunol.1000482 PMC3233222

[27] Yasuda K, Richez C, Maciaszek JW, Agrawal N, Akira S, et al (2007) Murine dendritic cell type I IFN production induced by human IgG-RNA immune complexes is IFN regulatory factor (IRF)5 and IRF7 dependent and is required for IL-6 production. J Immunol 178: 6876–6885. 17513736 10.4049/jimmunol.178.11.6876

[28] Paun A, Reinert JT, Jiang Z, Medin C, Balkhi MY, et al (2008) Functional characterization of murine interferon regulatory factor 5 (IRF-5) and its role in the innate antiviral response. J Biol Chem 283: 14295–14308. 18332133 10.1074/jbc.M800501200 PMC2386920

[29] Krausgruber T, Blazek K, Smallie T, Alzabin S, Lockstone H, et al (2011) IRF5 promotes inflammatory macrophage polarization and TH1-TH17 responses. Nat Immunol 12: 231–238. 21240265 10.1038/ni.1990

[30] Izaguirre A, Barnes BJ, Amrute S, Yeow WS, Megjugorac N, et al (2003) Comparative analysis of IRF and IFN-alpha expression in human plasmacytoid and monocyte-derived dendritic cells. J Leukoc Biol 74: 1125–1138. 12960254 10.1189/jlb.0603255

[31] Richez C, Yasuda K, Bonegio RG, Watkins AA, Aprahamian T, et al (2010) IFN regulatory factor 5 is required for disease development in the FcgammaRIIB−/−Yaa and FcgammaRIIB−/− mouse models of systemic lupus erythematosus. J Immunol 184: 796–806. 20007534 10.4049/jimmunol.0901748 PMC2858062

[32] Savitsky DA, Yanai H, Tamura T, Taniguchi T, Honda K (2010) Contribution of IRF5 in B cells to the development of murine SLE-like disease through its transcriptional control of the IgG2a locus. Proc Natl Acad Sci USA 107: 10154–10159. 20479222 10.1073/pnas.1005599107 PMC2890425

[33] Tada Y, Kondo S, Aoki S, Koarada S, Inoue H, et al (2011) Interferon regulatory factor 5 is critical for the development of lupus in MRL/lpr mice. Arthritis Rheum 63: 738–748. 21305501 10.1002/art.30183

[34] Shen H, Panchanathan R, Rajavelu P, Duan X, Gould KA, et al (2010) Gender-dependent expression of murine Irf5 gene: implications for sex bias in autoimmunity. J Mol Cell Biol 2: 284–290. 20802013 10.1093/jmcb/mjq023 PMC2952390

[35] Rullo OJ, Woo JM, Wu H, Hoftman AD, Maranian P, et al (2010) Association of IRF5 polymorphisms with activation of the interferon alpha pathway. Ann Rheum Dis 69: 611–617. 19854706 10.1136/ard.2009.118315 PMC3135414

[36] Kozyrev SV, Alarcon-Riquelme ME (2007) The genetics and biology of Irf5-mediated signaling in lupus. Autoimmunity 40: 591–601. 18075793 10.1080/08916930701510905

[37] Stone RC, Feng D, Deng J, Singh S, Yang L, et al (2012) Interferon regulatory factor 5 activation in monocytes of SLE patients is triggered by circulating autoantigens independent of type I IFN. Arthritis Rheum 64: 788–798. 21968701 10.1002/art.33395 PMC3288585

[38] Sambrook J, Russell DW (2001) Preparation of cDNA libraries and gene identification. In: Molecular Cloning: a laboratory manual. Cold Spring Harbor Laboratory Press, 11.69–11.70.

[39] Turro E, Su SY, Goncalves A, Coin LJ, Richardson S, et al (2011) Haplotype and isoform specific expression estimation using multi-mapping RNA-seq reads. Genome Biol 12: R13. 21310039 10.1186/gb-2011-12-2-r13 PMC3188795

[40] Wen F, Ellingson SM, Kyogoku C, Peterson EJ, Gaffney PM (2010) Exon 6 variants carried on systemic lupus erythematosus (SLE) risk haplotypes modulate IRF5 function. Autoimmunity 44: 82–89. 20695768 10.3109/08916934.2010.491842 PMC3104271

[41] Trapnell C, Pachter L, Salzberg SL (2009) TopHat: discovering splice junctions with RNA-Seq. Bioinformatics 25: 1105–1111. 19289445 10.1093/bioinformatics/btp120 PMC2672628

[42] Griffith M, Griffith OL, Mwenifumbo J, Goya R, Morrissy AS, et al (2010) Alternative expression analysis by RNA sequencing. Nature Methods 7: 843–847. 20835245 10.1038/nmeth.1503

[43] Lauwerys BR, Van Snick J, Houssiau FA (2002) Serum IL-12 in systemic lupus erythematosus: absence of p70 heterodimers but presence of p40 monomers correlating with disease activity. Lupus 11: 384–387. 12139377 10.1191/0961203302lu213oa

[44] Linker-Israeli M, Deans RJ, Wallace DJ, Prehn J, Ozeri-Chen T, et al (1991) Elevated levels of endogenous IL-6 in systemic lupus erythematosus. A putative role in pathogenesis. J Immunol 147: 117–123. 2051017

[45] Aringer M, Smolen JS (2003) SLE - Complex cytokine effects in a complex autoimmune disease: tumor necrosis factor in systemic lupus erythematosus. Arthritis Res Ther 5: 172–177. 12823847 10.1186/ar770 PMC165063

[46] Rogers S, Wells R, Rechsteiner M (1986) Amino acid sequences common to rapidly degraded proteins: the PEST hypothesis. Science 234: 364–368. 2876518 10.1126/science.2876518

[47] Shumway SD, Maki M, Miyamoto S (1999) The PEST domain of IkappaBalpha is necessary and sufficient for in vitro degradation by mu-calpain. J Biol Chem 274: 30874–30881. 10521480 10.1074/jbc.274.43.30874

[48] Jiang X, Coffino P, Li X (2004) Development of a method for screening short-lived proteins using green fluorescent protein. Genome Biol 5: R81. 15461799 10.1186/gb-2004-5-10-r81 PMC545601

[49] Corish P, Tyler-Smith C (1999) Attenuation of green fluorescent protein half-life in mammalian cells. Protein Eng 12: 1035–1040. 10611396 10.1093/protein/12.12.1035

[50] Mortazavi A, Williams BA, McCue K, Schaeffer L, Wold B (2008) Mapping and quantifying mammalian transcriptomes by RNA-Seq. Nat Methods 5: 621–628. 18516045 10.1038/nmeth.1226 PMC13303166

[51] Hiller D, Jiang H, Xu W, Wong W (2009) Identifiability of isoform deconvoluation from junction arrays and RNA-Seq. Bioinformatics 25: 3056–3059. 19762346 10.1093/bioinformatics/btp544 PMC3167695

[52] Jiang H, Wong WH (2009) Statistical inferences for isoform expression in RNA-Seq. Bioinformatics 25: 1026–1032. 19244387 10.1093/bioinformatics/btp113 PMC2666817

[53] Li B, Ruotti V, Stewart RM, Thomson JA, Dewey CN (2010) RNA-Seq gene expression estimation with read mapping uncertainty. Bioinformatics 26: 493–500. 20022975 10.1093/bioinformatics/btp692 PMC2820677

[54] Roberts A, Pimentel H, Trapnell C, Pachter L (2011) Identification of novel transcripts in annotated genomes using RNA-Seq. Bioinformatics 27: 2325–2329. 21697122 10.1093/bioinformatics/btr355

[55] Morley M, Molony CM, Weber TM, Devlin JL, Ewens KG, et al (2004) Genetic analysis of genome-wide variation in human gene expression. Nature 430: 743–747. 15269782 10.1038/nature02797 PMC2966974

[56] Alonso-Perez E, Suarez-Gestal M, Calaza M, Kwan T, Majewski J, et al (2011) *Cis*-regulation of IRF5 expression is unable to fully account for systemic lupuserythematosus association: analysis of multiple experiments with lymphoblastoid cell lines. Arthritis & Therap 13: R80. 10.1186/ar3343 PMC3218890 21627826

[57] Li H, Handsaker B, Wysoker A, Fennell T, Ruan J, et al (2009) The Sequence Alignment/Map format and SAMtools. Bioinformatics 25: 2078–2079. 19505943 10.1093/bioinformatics/btp352 PMC2723002

[58] Jarvinen TM, Hellquist A, Zucchelli M, Koskenmies S, Panelius J, et al (2012) Replication of genome-wide association study identified systemic lupus erythematosus susceptibility genes affirms B-cell receptor pathway signalling and strengthens the role of IRF5 in disease susceptibility in a Northern European population. Rheumatology 51: 87–92. 22039224 10.1093/rheumatology/ker263

[59] Barnes BJ, Field A, Pitha-Rowe PM (2003) Virus-induced heterodimer formation between IRF-5 and IRF-7 modulates assembly of the IFNA enhanceosome in vivo and transcriptional activity of IFNA genes. J Biol Chem 278: 16630–16641. 12600985 10.1074/jbc.M212609200

[60] Cheng TF, Brzostek S, Ando O, Van Scoy S, Kumar KP, et al (2006) Differential activation of IFN regulatory factor (IRF)-3 and IRF-5 transcription factors during viral infection. J Immunol 176: 7462–7470. 16751392 10.4049/jimmunol.176.12.7462

[61] Langmead B (2010) Aligning short sequencing reads with Bowtie. Curr Protoc Bioinformatics Chapter 11, Unit 11 7. 10.1002/0471250953.bi1107s32 PMC3010897 21154709

[62] Langmead B, Trapnell C, Pop M, Salzberg SL (2009) Ultrafast and memory-efficient alignment of short DNA sequences to the human genome. Genome Biol 10: R25. 19261174 10.1186/gb-2009-10-3-r25 PMC2690996

[63] Burset M, Seledtsov IA, Solovyev VV (2000) Analysis of canonical and non-canonical splice sites in mammalian genomes. Nucl Acids Res 28: 4364–4375. 11058137 10.1093/nar/28.21.4364 PMC113136

[64] Houseley J, Tollervey D (2010) Apparent non-canonical trans-splicing is generated by reverse transcriptase *in vitro* . PLoS ONE 5(8): e12271. 20805885 10.1371/journal.pone.0012271 PMC2923612

[65] Robinson JY, Thorvaldsdóttir H, Winckler W, Guttman M, Lander ES, et al (2011) Integrative Genomics Viewer. Nature Biotechnology 29: 24–26. 10.1038/nbt.1754 PMC3346182 21221095

