# Supplementary figures and images for: RNA-Seq for Enrichment and Analysis of IRF5 Transcript Expression in SLE
Source: PLoS One. 2013 Jan 18;8(1):e54487. doi: 10.1371/journal.pone.0054487 (PMC3548774; doi:10.1371/journal.pone.0054487)

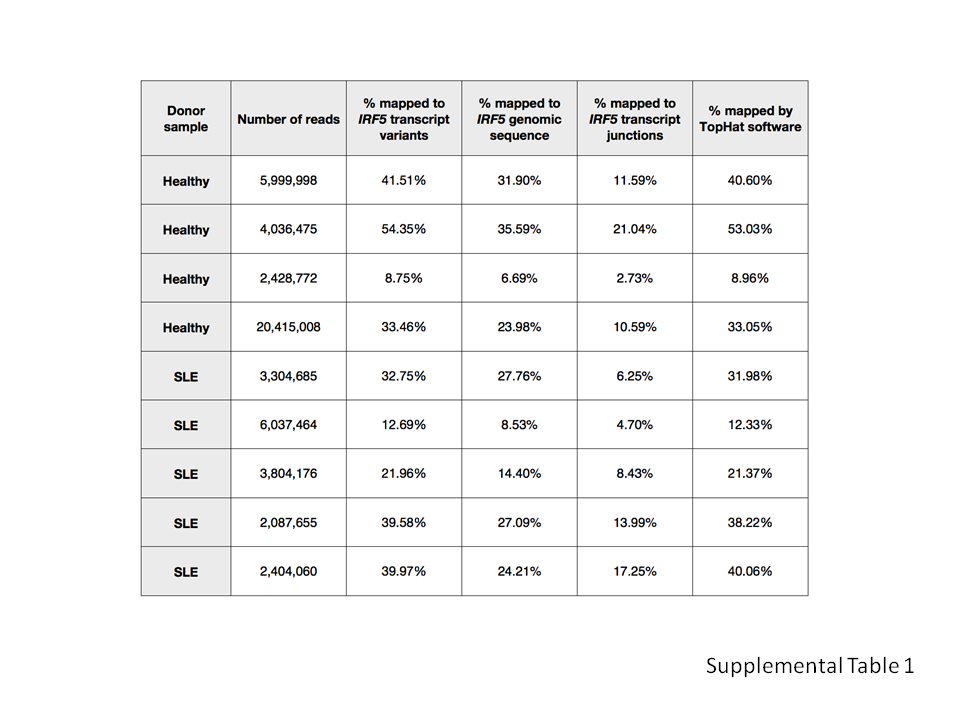

Supplement: Table S1 — Read mapping statistics. For each sequenced healthy donor and SLE patient sample, the number of reads as well as percentages of reads mapped within each Bowtie alignment is shown. For MMSeq-based expression estimates, mapping was performed to transcript variants (column 3); to IRF5 genomic sequence for pile-up views (column 4); to junction sequences for junction-based expression estimates (column 5); and iteratively to IRF5 and its predicted junctions by TopHat de novo junction detection software (column 6). (TIF) [file pone.0054487.s002.tif]

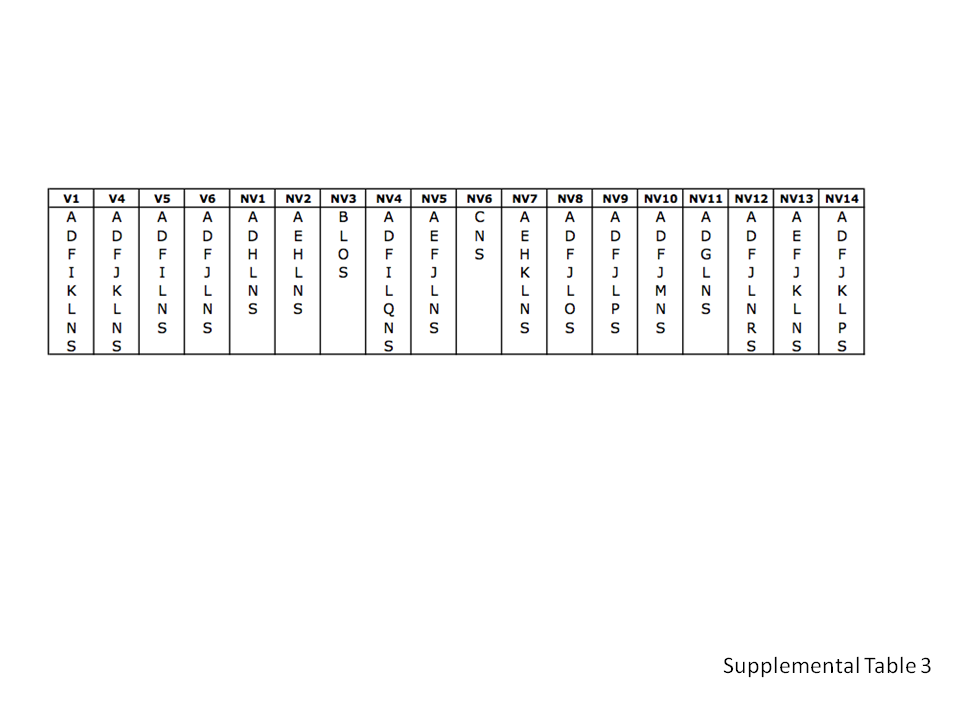

Supplement: Table S3 — List of junctions contained in each full-length alternatively spliced IRF5 transcript. Each of the 18 transcripts contains a unique combination of junctions. (TIF) [file pone.0054487.s004.tif]

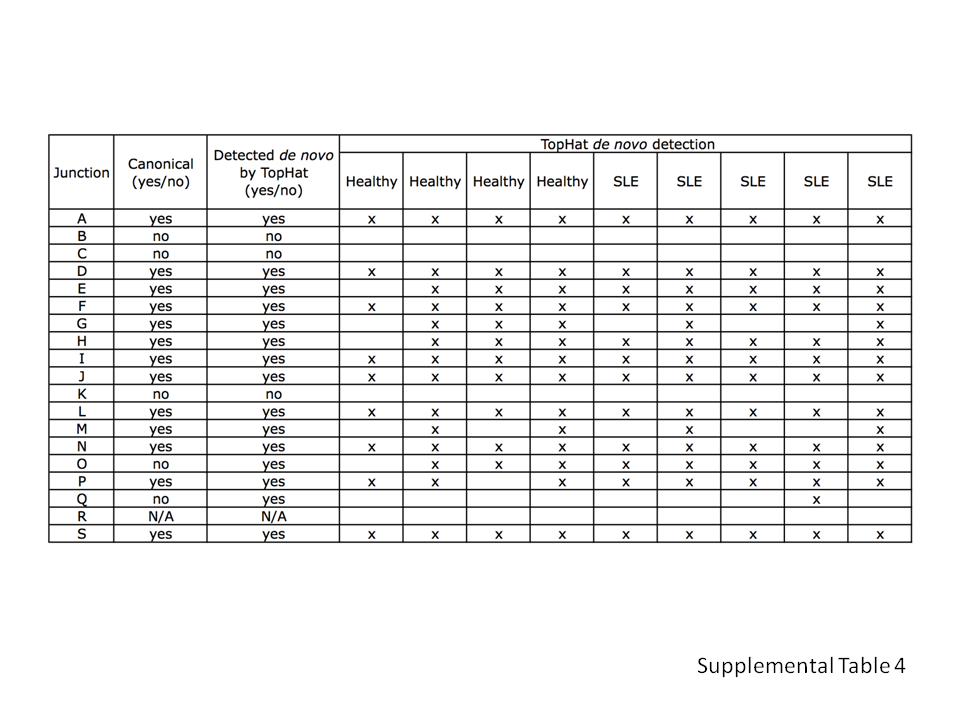

Supplement: Table S4 — List of junctions in the IRF5 variant transcriptome that were detected by TopHat. Junctions were considered canonical if splice sites consisted of nucleotides GT…AG, GC…AG, AT…AC, GG…AG, GT…TG, GT…GG, or CT…AG [62]. Individual junctions present in each sample are shown by the x. (TIF) [file pone.0054487.s005.tif]

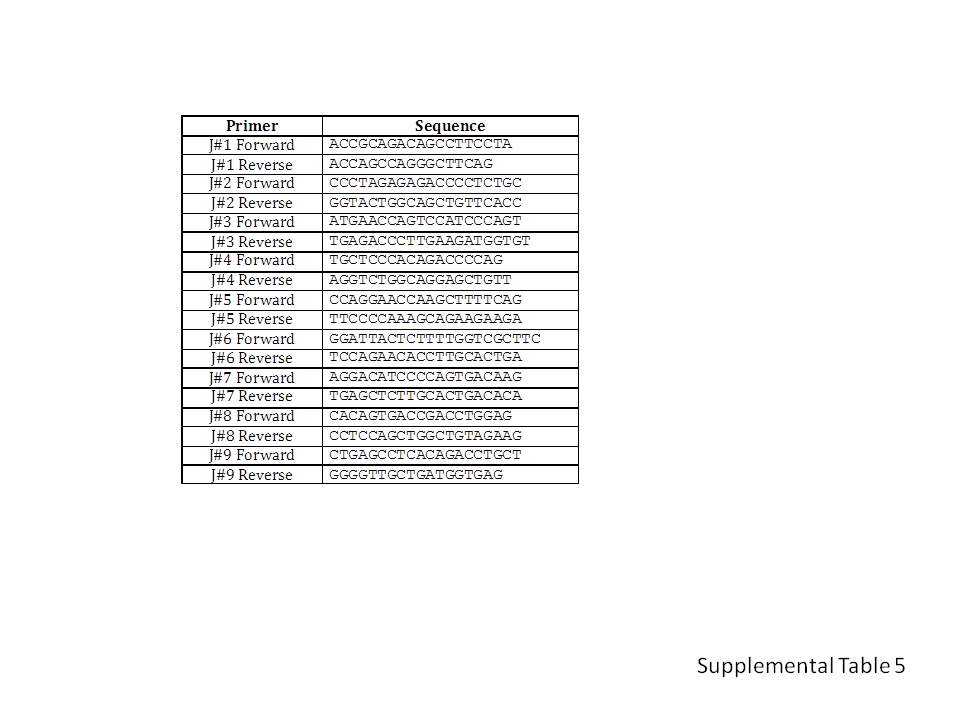

Supplement: Table S5 — List of primers used to confirm TopHat discovered junctions 1–9. (TIF) [file pone.0054487.s006.tif]

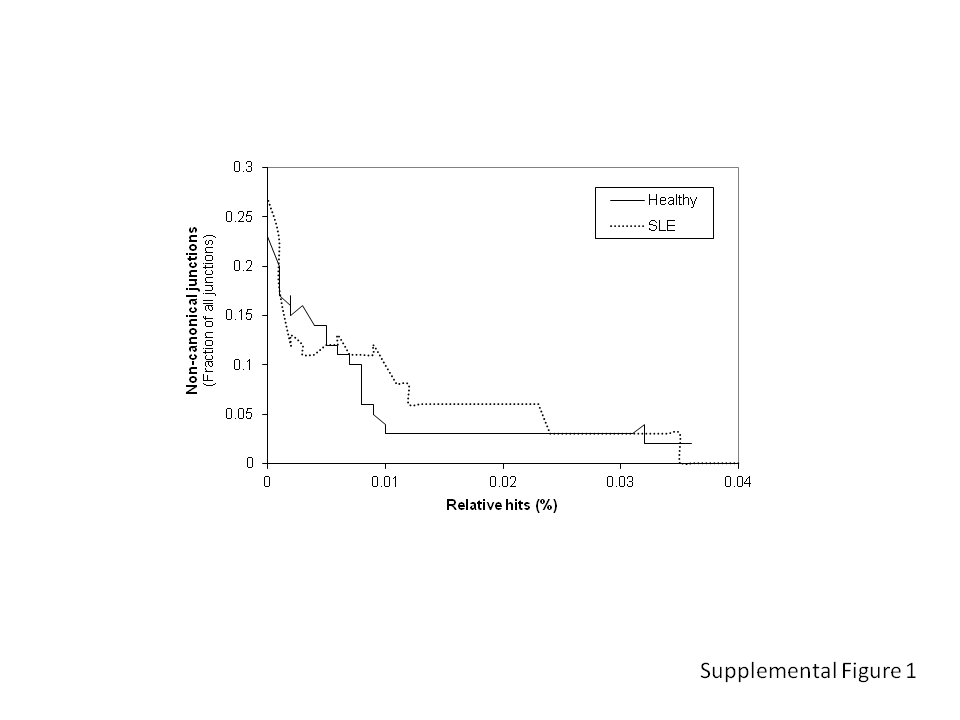

Supplement: Method S1 — Filtering de novo junctions reported by TopHat to exclude random splicing. True splice junctions are more likely to have a canonical or near canonical splice site, while random (non-significant) splicing is more likely to use non-canonical splice sites [62]–[63]; thus the fraction of non-canonical sites among junctions reported can serve as an indicator of the false positive rate. Data in Table S2 reveal that the majority of junction splice sites found in the IRF5 variant transcriptome feature the canonical dinucleotides GT and AG as intronic donor and acceptor splice sites, respectively. However, well-known IRF5 variants V1 and V4 contain the non-canonical junction K that generates one of the Ex6 deletions [19]. Thus, validity of junctions discovered de novo might better be determined with a cut-off. When we define “relative hits” as the number of reads mapping to a TopHat-reported junction per fraction of all reads for all reported junctions, we observe that the fraction of non-canonical splice sites for discovered junctions decreases when the number of relative hits to the junctions increase (Figure S1). When the relative hits for a junction reaches 0.01%, the frequency of having a non-canonical splicing site tends to level off to near zero. Based on these findings, we set a threshold of 0.01% and discard reported junctions with relative hits below this value, thus more likely including junctions that represent true, non-random splicing. (TIF) [file pone.0054487.s009.tif]
