# Supplementary material for: RNA-Seq for Enrichment and Analysis of IRF5 Transcript Expression in SLE
Source: PLoS One. 2013 Jan 18;8(1):e54487. doi: 10.1371/journal.pone.0054487 (PMC3548774; doi:10.1371/journal.pone.0054487)
Supplement: Dataset S1 — Sequences of the 14 novel alternatively spliced IRF5 transcripts. New variants were identified by cloning from purified monocytes of healthy donors and SLE patients. (DOC) [file pone.0054487.s007.doc]

**Supporting Dataset 1.** Sequences of the 14 novel alternatively spliced *IRF5* transcripts identified by cloning from purified monocytes of healthy donors and SLE patients.

> NV1

GCCCGGCAGGTTGGCGGACCGGCGGGAGGCGCAGCCTGGGCAGAGCTCAGCTTGGTCCCG

CCGCCCGGCCGGTGCTCCCTGGCGCAGCCACGCAGGCGCACCGCAGACAGACCCCTCTGC

CATGAACCAGTCCATCCCAGTGGCTCCCACCCCACCCCGCCGCGTGCGGCTGAAGCCCTG

GCTGGTGGCCCAGGTGAACAGCTGCCAGTACCCAGGGCTTCAATGGGTCAACGGGGAAAA

GAAATTATTCTGCATCCCCTGGAGGCATGCCACAAGGCATGGTCCCAGCCAGGACGGAGA

TAACACCATCTTCAAGGCCTGGGCCAAGGAGACAGGGAAATACACCGAAGGCGTGGATGA

AGCCGATCCGGCCAAGTGGAAGGCCAACCTGCGCTGTGCCCTTAACAAGAGCCGGGACTT

CCGCCTCATCTACGACGGGCCCCGGGACATGCCACCTCAGCCCTACAAGATCTACGAGGT

CTGCTCCAATGGCCCTGCTCCCACAGACTCCCAGCCCCCTGAGGATTACTCTTTTGGTGC

AGGAGAGGAGGAGGAAGAAGAGGAAGAGAGGATGTCAAGTGGCCGCCCACTCTGCAGCCG

CCCACTCTGCGGCCGCCTACTCTGCAGCCGCCCACTCTGCAGCCGCCCGTGGTGCTGGGT

CCCCCTGCTCCAGACCCCAGCCCCCTGGCTCCTCCCCCTGGCAACCCTGCTGGCTTCAGG

GAGCTTCTCTCTGAGGTCCTGGAGCCTGGGCCCCTGCCTGCCAGCCTGCCCCCTGCAGGC

GAACAGCTCCTGCCAGACCTGCTGATCAGCCCCCACATGCTGCCTCTGACCGACCTGGAG

ATCAAGTTTCAGTACCGGGGGCGGCCACCCCGGGCCCTCACCATCAGCAACCCCCATGGC

TGCCGGCTCTTCTACAGCCAGCTGGAGGCCACCCAGGAGCAGGTGGAACTCTTCGGCCCC

ATAAGCCTGGAGCAAGTGCGCTTCCCCAGCCCTGAGGACATCCCCAGTGACAAGCAGCGC

TTCTACACGAACCAGCTGCTGGATGTCCTGGACCGCGGGCTCATCCTCCAGCTACAGGGC

CAGGACCTTTATGCCATCCGCCTGTGTCAGTGCAAGGTGTTCTGGAGCGGGCCTTGTGCC

TCAGCCCATGACTCATGCCCCAACCCCATCCAGCGGGAGGTCAAGACCAAGCTTTTCAGC

CTGGAGCATTTTCTCAATGAGCTCATCCTGTTCCAAAAGGGCCAGACCAACACCCCACCA

CCCTTCGAGATCTTCTTCTGCTTTGGGGAAGAATGGCCTGACCGCAAACCCCGAGAGAAG

AAGCTCATTACTGTACAGGTGGTGCCTGTAGCAGCTCGACTGCTGCTGGAGATGTTCTCA

GGGGAGCTATCTTGGTCAGCTGATAGTATCCGGCTACAGATCTCAAACCCAGACCTCAAA

GACCGCATGGTGGAGCAATTCAAGGAGCTCCATCACATCTGGCAGTCCCAGCAGCGGTTG

CAGCCTGTGGCCCAGGCCCCTCCTGGAGCAGGCCTTGGTGTTGGCCAGGGGCCCTGGCCT

ATGCACCCAGCTGGCATGCAATAACAAGGCTGCAGACGGTGACTGGCCCTGGCTTCCTGG

GTGGCGGTGCGGACTGATGTGGAGATGTGACAGCCCCGATGAGCACCTGGCTGGCTGCAG

GGTCCTACCTCTGGGTTTCCTGGAAGTGGATTTGGGCCAAGAAGGAGAGGGAGAAAGGCC

CGAGCCCCTGCCTTCCCGGGCCTTTCTCTCCTGGGCTGTCTCTGGTCTGGTCAGCCTGGC

TCTCGGGAAATTCAGCCATGAGCAGGGAAAGAACTCTCCCAACCCTGGGGCCTAGCTGTA

TAGGAGGAATTGCCTAAGGGTGGCCCACTCTTGTGATTGCCCCATTTCCTCTGGCAACAA

AAGCCAGAGTGTTGTGGGCCAAGTCCCCCCACAGGGCCTCTGCAGGGCATGGCCCTGATT

TCCCTGGTTTGAGACTCACTTCCTCATCTCCCTGTCCTCTGAGATAATATGAGTGAGCAC

TTAGGTATCATATCAGATGCTCAAGGCTGGCAGCTACCCCCTTCTTGAGAGTCCAAGAAC

CTGGAGCAGAAATAATTTTTATGTATTTTTGGATTAATGAATGTTAAAAACAGACTCAGC

TGTTTCTTTCCTTTTACTACTACCAGTTGCTCCCATGCTGCTCCACCAGGCCCTGTTTCG

GATGCCAACTGGCCCACTCCCCAAGCACTTGCCCCCAGCTTGCGACCATTGGCACTGGGA

GGGCCTGGCTTCTGGGCTGATGGGTCAGTTGGGCCTTCATAAACACTCACCTGGCTGGCT

TTGCCTTCCAGGAGGAAGCTGGCTGAAGCAAGGGTGTGGAATTTTAAATGTGTGCACAGT

CTGGAAAACTGTCAGAATCAGTTTTCCCATAAAAGGGTGGGCTAGCATTGCAGCTGCATT

TGGGACCATTCAAATCTGTCACTCTCTTGTGTATATTCCTGTGCTATTAAATATATCAGG

GCAGTGCATGTAAATCATCCTGATATATTTAATATATTTATTATATTGTCCCCCGAGGTG

GGGACAGTGAGTGAGTTCTCTTAGTCCCCCCAGAGCTGGTTGTTAAAGAGCCTGGCACCT

ACCCGCTCTCACTTCATCTGTGTCATCTCTGCACACTCCAGCCCACTTTCTGCCTTCAGC

CATTGAGTGGAAGCTGCCCCAGGCCCTTACCAGGTGCAGATGCCCAATCTTGATGCCCAG

CCATCAGAACTGTGAGCCAAATAAACCTTTTTCTGTATAAA

>NV2

GCCCGGCAGGTTGGCGGACCGGCGGGAGGCGCAGCCTGGGCAGAGCTCAGCTTGGTCCCG

CCGCCCGGCCGGTGCTCCCTGGCGCAGCCACGCAGGCGCACCGCAGACAGACCCCTCTGC

CATGAACCAGTCCATCCCAGTGGCTCCCACCCCACCCCGCCGCGTGCGGCTGAAGCCCTG

GCTGGTGGCCCAGGTGAACAGCTGCCAGTACCCAGGGCTTCAATGGGTCAACGGGGAAAA

GAAATTATTCTGCATCCCCTGGAGGCATGCCACAAGGCATGGTCCCAGCCAGGACGGAGA

TAACACCATCTTCAAGGCCTGGGCCAAGGAGACAGGGAAATACACCGAAGGCGTGGATGA

AGCCGATCCGGCCAAGTGGAAGGCCAACCTGCGCTGTGCCCTTAACAAGAGCCGGGACTT

CCGCCTCATCTACGACGGGCCCCGGGACATGCCACCTCAGCCCTACAAGATCTACGAGAC

TCCCAGCCCCCTGAGGATTACTCTTTTGGTGCAGGAGAGGAGGAGGAAGAAGAGGAAGAG

AGGATGTCAAGTGGCCGCCCACTCTGCAGCCGCCCACTCTGCGGCCGCCTACTCTGCAGC

CGCCCACTCTGCAGCCGCCCGTGGTGCTGGGTCCCCCTGCTCCAGACCCCAGCCCCCTGG

CTCCTCCCCCTGGCAACCCTGCTGGCTTCAGGGAGCTTCTCTCTGAGGTCCTGGAGCCTG

GGCCCCTGCCTGCCAGCCTGCCCCCTGCAGGCGAACAGCTCCTGCCAGACCTGCTGATCA

GCCCCCACATGCTGCCTCTGACCGACCTGGAGATCAAGTTTCAGTACCGGGGGCGGCCAC

CCCGGGCCCTCACCATCAGCAACCCCCATGGCTGCCGGCTCTTCTACAGCCAGCTGGAGG

CCACCCAGGAGCAGGTGGAACTCTTCGGCCCCATAAGCCTGGAGCAAGTGCGCTTCCCCA

GCCCTGAGGACATCCCCAGTGACAAGCAGCGCTTCTACACGAACCAGCTGCTGGATGTCC

TGGACCGCGGGCTCATCCTCCAGCTACAGGGCCAGGACCTTTATGCCATCCGCCTGTGTC

AGTGCAAGGTGTTCTGGAGCGGGCCTTGTGCCTCAGCCCATGACTCATGCCCCAACCCCA

TCCAGCGGGAGGTCAAGACCAAGCTTTTCAGCCTGGAGCATTTTCTCAATGAGCTCATCC

TGTTCCAAAAGGGCCAGACCAACACCCCACCACCCTTCGAGATCTTCTTCTGCTTTGGGG

AAGAATGGCCTGACCGCAAACCCCGAGAGAAGAAGCTCATTACTGTACAGGTGGTGCCTG

TAGCAGCTCGACTGCTGCTGGAGATGTTCTCAGGGGAGCTATCTTGGTCAGCTGATAGTA

TCCGGCTACAGATCTCAAACCCAGACCTCAAAGACCGCATGGTGGAGCAATTCAAGGAGC

TCCATCACATCTGGCAGTCCCAGCAGCGGTTGCAGCCTGTGGCCCAGGCCCCTCCTGGAG

CAGGCCTTGGTGTTGGCCAGGGGCCCTGGCCTATGCACCCAGCTGGCATGCAATAACAAG

GCTGCAGACGGTGACTGGCCCTGGCTTCCTGGGTGGCGGTGCGGACTGATGTGGAGATGT

GACAGCCCCGATGAGCACCTGGCTGGCTGCAGGGTCCTACCTCTGGGTTTCCTGGAAGTG

GATTTGGGCCAAGAAGGAGAGGGAGAAAGGCCCGAGCCCCTGCCTTCCCGGGCCTTTCTC

TCCTGGGCTGTCTCTGGTCTGGTCAGCCTGGCTCTCGGGAAATTCAGCCATGAGCAGGGA

AAGAACTCTCCCAACCCTGGGGCCTAGCTGTATAGGAGGAATTGCCTAAGGGTGGCCCAC

TCTTGTGATTGCCCCATTTCCTCTGGCAACAAAAGCCAGAGTGTTGTGGGCCAAGTCCCC

CCACAGGGCCTCTGCAGGGCATGGCCCTGATTTCCCTGGTTTGAGACTCACTTCCTCATC

TCCCTGTCCTCTGAGATAATATGAGTGAGCACTTAGGTATCATATCAGATGCTCAAGGCT

GGCAGCTACCCCCTTCTTGAGAGTCCAAGAACCTGGAGCAGAAATAATTTTTATGTATTT

TTGGATTAATGAATGTTAAAAACAGACTCAGCTGTTTCTTTCCTTTTACTACTACCAGTT

GCTCCCATGCTGCTCCACCAGGCCCTGTTTCGGATGCCAACTGGCCCACTCCCCAAGCAC

TTGCCCCCAGCTTGCGACCATTGGCACTGGGAGGGCCTGGCTTCTGGGCTGATGGGTCAG

TTGGGCCTTCATAAACACTCACCTGGCTGGCTTTGCCTTCCAGGAGGAAGCTGGCTGAAG

CAAGGGTGTGGAATTTTAAATGTGTGCACAGTCTGGAAAACTGTCAGAATCAGTTTTCCC

ATAAAAGGGTGGGCTAGCATTGCAGCTGCATTTGGGACCATTCAAATCTGTCACTCTCTT

GTGTATATTCCTGTGCTATTAAATATATCAGGGCAGTGCATGTAAATCATCCTGATATAT

TTAATATATTTATTATATTGTCCCCCGAGGTGGGGACAGTGAGTGAGTTCTCTTAGTCCC

CCCAGAGCTGGTTGTTAAAGAGCCTGGCACCTACCCGCTCTCACTTCATCTGTGTCATCT

CTGCACACTCCAGCCCACTTTCTGCCTTCAGCCATTGAGTGGAAGCTGCCCCAGGCCCTT

ACCAGGTGCAGATGCCCAATCTTGATGCCCAGCCATCAGAACTGTGAGCCAAATAAACCT

TTTTCTGTATAAA

>NV3

GCCCGGCAGGTTGGCGGACCGGCGGGAGGCGCAGCCTGGGCAGAGCTCAGCTTGGTCCCG

CCGCCCGGCCGGTGCTCCCTGGCGCAGCCACGCAGGCGCACCGCAGACAGACCCCTCTGC

CATGAACCAGTCCATCCCAGTGGCTCCCACCCCACCCCGCCGCGTGCGGCTGAAGCCCTG

GCTGGTGGCCCAGGTGAACAGCTGCCAGTACCCAGGGCTTCAATGGGTCAACGGGGAAAA

GAAATTATTCTGCATCCCCTGGAGGCATGCCACACTCTGCAGCCGCCCACTCTGCAGCCG

CCCGTGGTGCTGGGTCCCCCTGCTCCAGACCCCAGCCCCCTGGCTCCTCCCCCTGGCAAC

CCTGCTGGCTTCAGGGAGCTTCTCTCTGAGGTCCTGGAGCCTGGGCCCCTGCCTGCCAGC

CTGCCCCCTGCAGGCGAACAGCTCCTGCCAGACCTGCTGATCAGCCCCCACATGCTGCCT

CTGACCGACCTGGAGATCAAGTTTCAGTACCGGGGGCGGCCACCCCGGGCCCTCACCATC

AGCAACCCCCATGGCTGCCGGCTCTTCTACAGCCAGCTGGAGGCCACCCAGGAGCAGGTG

GAACTCTTCGGCCCCATAAGCCTGGAGCAAGTGCGCTTCCCCAGCCCTGAGGACATCCCC

AGTGACAAGCAGCGCTTCTACACGAACCAGCTGCTGGATGTCCTGGACCGCGGGCTCATC

CTCCAGCTACAGGGCCAGGACCTTTATGCCATCCGCCTGTGTCAGTGCAAGGTGTTCTGG

AGCGGGCCTTGTGCCTCAGCCCATGACTCATGCCCCAACCCCATCCAGCGGGAGGTCAAG

ACCAAGCTTTTCAGCCTGGAGCATTTTCTCAATGGGGCCAGACCAACACCCCACCACCCT

TCGAGATCTTCTTCTGCTTTGGGGAAGAATGGCCTGACCGCAAACCCCGAGAGAAGAAGC

TCATTACTGTACAGGTGGTGCCTGTAGCAGCTCGACTGCTGCTGGAGATGTTCTCAGGGG

AGCTATCTTGGTCAGCTGATAGTATCCGGCTACAGATCTCAAACCCAGACCTCAAAGACC

GCATGGTGGAGCAATTCAAGGAGCTCCATCACATCTGGCAGTCCCAGCAGCGGTTGCAGC

CTGTGGCCCAGGCCCCTCCTGGAGCAGGCCTTGGTGTTGGCCAGGGGCCCTGGCCTATGC

ACCCAGCTGGCATGCAATAACAAGGCTGCAGACGGTGACTGGCCCTGGCTTCCTGGGTGG

CGGTGCGGACTGATGTGGAGATGTGACAGCCCCGATGAGCACCTGGCTGGCTGCAGGGTC

CTACCTCTGGGTTTCCTGGAAGTGGATTTGGGCCAAGAAGGAGAGGGAGAAAGGCCCGAG

CCCCTGCCTTCCCGGGCCTTTCTCTCCTGGGCTGTCTCTGGTCTGGTCAGCCTGGCTCTC

GGGAAATTCAGCCATGAGCAGGGAAAGAACTCTCCCAACCCTGGGGCCTAGCTGTATAGG

AGGAATTGCCTAAGGGTGGCCCACTCTTGTGATTGCCCCATTTCCTCTGGCAACAAAAGC

CAGAGTGTTGTGGGCCAAGTCCCCCCACAGGGCCTCTGCAGGGCATGGCCCTGATTTCCC

TGGTTTGAGACTCACTTCCTCATCTCCCTGTCCTCTGAGATAATATGAGTGAGCACTTAG

GTATCATATCAGATGCTCAAGGCTGGCAGCTACCCCCTTCTTGAGAGTCCAAGAACCTGG

AGCAGAAATAATTTTTATGTATTTTTGGATTAATGAATGTTAAAAACAGACTCAGCTGTT

TCTTTCCTTTTACTACTACCAGTTGCTCCCATGCTGCTCCACCAGGCCCTGTTTCGGATG

CCAACTGGCCCACTCCCCAAGCACTTGCCCCCAGCTTGCGACCATTGGCACTGGGAGGGC

CTGGCTTCTGGGCTGATGGGTCAGTTGGGCCTTCATAAACACTCACCTGGCTGGCTTTGC

CTTCCAGGAGGAAGCTGGCTGAAGCAAGGGTGTGGAATTTTAAATGTGTGCACAGTCTGG

AAAACTGTCAGAATCAGTTTTCCCATAAAAGGGTGGGCTAGCATTGCAGCTGCATTTGGG

ACCATTCAAATCTGTCACTCTCTTGTGTATATTCCTGTGCTATTAAATATATCAGGGCAG

TGCATGTAAATCATCCTGATATATTTAATATATTTATTATATTGTCCCCCGAGGTGGGGA

CAGTGAGTGAGTTCTCTTAGTCCCCCCAGAGCTGGTTGTTAAAGAGCCTGGCACCTACCC

GCTCTCACTTCATCTGTGTCATCTCTGCACACTCCAGCCCACTTTCTGCCTTCAGCCATT

GAGTGGAAGCTGCCCCAGGCCCTTACCAGGTGCAGATGCCCAATCTTGATGCCCAGCCAT

CAGAACTGTGAGCCAAATAAACCTTTTTCTGTATAAA

> NV4

GCCCGGCAGGTTGGCGGACCGGCGGGAGGCGCAGCCTGGGCAGAGCTCAGCTTGGTCCCG

CCGCCCGGCCGGTGCTCCCTGGCGCAGCCACGCAGGCGCACCGCAGACAGACCCCTCTGC

CATGAACCAGTCCATCCCAGTGGCTCCCACCCCACCCCGCCGCGTGCGGCTGAAGCCCTG

GCTGGTGGCCCAGGTGAACAGCTGCCAGTACCCAGGGCTTCAATGGGTCAACGGGGAAAA

GAAATTATTCTGCATCCCCTGGAGGCATGCCACAAGGCATGGTCCCAGCCAGGACGGAGA

TAACACCATCTTCAAGGCCTGGGCCAAGGAGACAGGGAAATACACCGAAGGCGTGGATGA

AGCCGATCCGGCCAAGTGGAAGGCCAACCTGCGCTGTGCCCTTAACAAGAGCCGGGACTT

CCGCCTCATCTACGACGGGCCCCGGGACATGCCACCTCAGCCCTACAAGATCTACGAGGT

CTGCTCCAATGGCCCTGCTCCCACAGACTCCCAGCCCCCTGAGGATTACTCTTTTGGTGC

AGGAGAGGAGGAGGAAGAAGAGGAAGAGCTGCAGAGGATGTTGCCAAGCCTGAGCCTCAC

AGATGCAGTGCAGTCTGGCCCCCACATGACACCCTATTCTTTACTCAAAGAGGATGTCAA

GTGGCCGCCCACTCTGCAGCCGCCCACTCTGCGGCCGCCTACTCTGCAGCCGCCCACTCT

GCAGCCGCCCGTGGTGCTGGGTCCCCCTGCTCCAGACCCCAGCCCCCTGGCTCCTCCCCC

TGGCAACCCTGCTGGCTTCAGGGAGCTTCTCTCTGAGGTCCTGGAGCCTGGGCCCCTGCC

TGCCAGCCTGCCCCCTGCAGGCGAACAGCTCCTGCCAGACCTGCTGATCAGCCCCCACAT

GCTGCCTCTGACCGACCTGGAGATCAAGTTTCAGTACCGGGGGCGGCCACCCCGGGCCCT

CACCATCAGCAACCCCCATGGCTGCCGGCTCTTCTACAGCCAGCTGGAGGCCACCCAGGA

GCAGGTGGAACTCTTCGGCCCCATATGCCATCCGCCTGTGTCAGTGCAAGGTGTTCTGGA

GCGGGCCTTGTGCCTCAGCCCATGACTCATGCCCCAACCCCATCCAGCGGGAGGTCAAGA

CCAAGCTTTTCAGCCTGGAGCATTTTCTCAATGAGCTCATCCTGTTCCAAAAGGGCCAGA

CCAACACCCCACCACCCTTCGAGATCTTCTTCTGCTTTGGGGAAGAATGGCCTGACCGCA

AACCCCGAGAGAAGAAGCTCATTACTGTACAGGTGGTGCCTGTAGCAGCTCGACTGCTGC

TGGAGATGTTCTCAGGGGAGCTATCTTGGTCAGCTGATAGTATCCGGCTACAGATCTCAA

ACCCAGACCTCAAAGACCGCATGGTGGAGCAATTCAAGGAGCTCCATCACATCTGGCAGT

CCCAGCAGCGGTTGCAGCCTGTGGCCCAGGCCCCTCCTGGAGCAGGCCTTGGTGTTGGCC

AGGGGCCCTGGCCTATGCACCCAGCTGGCATGCAATAACAAGGCTGCAGACGGTGACTGG

CCCTGGCTTCCTGGGTGGCGGTGCGGACTGATGTGGAGATGTGACAGCCCCGATGAGCAC

CTGGCTGGCTGCAGGGTCCTACCTCTGGGTTTCCTGGAAGTGGATTTGGGCCAAGAAGGA

GAGGGAGAAAGGCCCGAGCCCCTGCCTTCCCGGGCCTTTCTCTCCTGGGCTGTCTCTGGT

CTGGTCAGCCTGGCTCTCGGGAAATTCAGCCATGAGCAGGGAAAGAACTCTCCCAACCCT

GGGGCCTAGCTGTATAGGAGGAATTGCCTAAGGGTGGCCCACTCTTGTGATTGCCCCATT

TCCTCTGGCAACAAAAGCCAGAGTGTTGTGGGCCAAGTCCCCCCACAGGGCCTCTGCAGG

GCATGGCCCTGATTTCCCTGGTTTGAGACTCACTTCCTCATCTCCCTGTCCTCTGAGATA

ATATGAGTGAGCACTTAGGTATCATATCAGATGCTCAAGGCTGGCAGCTACCCCCTTCTT

GAGAGTCCAAGAACCTGGAGCAGAAATAATTTTTATGTATTTTTGGATTAATGAATGTTA

AAAACAGACTCAGCTGTTTCTTTCCTTTTACTACTACCAGTTGCTCCCATGCTGCTCCAC

CAGGCCCTGTTTCGGATGCCAACTGGCCCACTCCCCAAGCACTTGCCCCCAGCTTGCGAC

CATTGGCACTGGGAGGGCCTGGCTTCTGGGCTGATGGGTCAGTTGGGCCTTCATAAACAC

TCACCTGGCTGGCTTTGCCTTCCAGGAGGAAGCTGGCTGAAGCAAGGGTGTGGAATTTTA

AATGTGTGCACAGTCTGGAAAACTGTCAGAATCAGTTTTCCCATAAAAGGGTGGGCTAGC

ATTGCAGCTGCATTTGGGACCATTCAAATCTGTCACTCTCTTGTGTATATTCCTGTGCTA

TTAAATATATCAGGGCAGTGCATGTAAATCATCCTGATATATTTAATATATTTATTATAT

TGTCCCCCGAGGTGGGGACAGTGAGTGAGTTCTCTTAGTCCCCCCAGAGCTGGTTGTTAA

AGAGCCTGGCACCTACCCGCTCTCACTTCATCTGTGTCATCTCTGCACACTCCAGCCCAC

TTTCTGCCTTCAGCCATTGAGTGGAAGCTGCCCCAGGCCCTTACCAGGTGCAGATGCCCA

ATCTTGATGCCCAGCCATCAGAACTGTGAGCCAAATAAACCTTTTTCTGTATAAA

>NV5

GCCCGGCAGGTTGGCGGACCGGCGGGAGGCGCAGCCTGGGCAGAGCTCAGCTTGGTCCCG

CCGCCCGGCCGGTGCTCCCTGGCGCAGCCACGCAGGCGCACCGCAGACAGACCCCTCTGC

CATGAACCAGTCCATCCCAGTGGCTCCCACCCCACCCCGCCGCGTGCGGCTGAAGCCCTG

GCTGGTGGCCCAGGTGAACAGCTGCCAGTACCCAGGGCTTCAATGGGTCAACGGGGAAAA

GAAATTATTCTGCATCCCCTGGAGGCATGCCACAAGGCATGGTCCCAGCCAGGACGGAGA

TAACACCATCTTCAAGGCCTGGGCCAAGGAGACAGGGAAATACACCGAAGGCGTGGATGA

AGCCGATCCGGCCAAGTGGAAGGCCAACCTGCGCTGTGCCCTTAACAAGAGCCGGGACTT

CCGCCTCATCTACGACGGGCCCCGGGACATGCCACCTCAGCCCTACAAGATCTACGAGAC

TCCCAGCCCCCTGAGGATTACTCTTTTGGTGCAGGAGAGGAGGAGGAAGAAGAGGAAGAG

CTGCAGAGGATGTTGCCAAGCCTGAGCCTCACAGAGGATGTCAAGTGGCCGCCCACTCTG

CAGCCGCCCACTCTGCGGCCGCCTACTCTGCAGCCGCCCACTCTGCAGCCGCCCGTGGTG

CTGGGTCCCCCTGCTCCAGACCCCAGCCCCCTGGCTCCTCCCCCTGGCAACCCTGCTGGC

TTCAGGGAGCTTCTCTCTGAGGTCCTGGAGCCTGGGCCCCTGCCTGCCAGCCTGCCCCCT

GCAGGCGAACAGCTCCTGCCAGACCTGCTGATCAGCCCCCACATGCTGCCTCTGACCGAC

CTGGAGATCAAGTTTCAGTACCGGGGGCGGCCACCCCGGGCCCTCACCATCAGCAACCCC

CATGGCTGCCGGCTCTTCTACAGCCAGCTGGAGGCCACCCAGGAGCAGGTGGAACTCTTC

GGCCCCATAAGCCTGGAGCAAGTGCGCTTCCCCAGCCCTGAGGACATCCCCAGTGACAAG

CAGCGCTTCTACACGAACCAGCTGCTGGATGTCCTGGACCGCGGGCTCATCCTCCAGCTA

CAGGGCCAGGACCTTTATGCCATCCGCCTGTGTCAGTGCAAGGTGTTCTGGAGCGGGCCT

TGTGCCTCAGCCCATGACTCATGCCCCAACCCCATCCAGCGGGAGGTCAAGACCAAGCTT

TTCAGCCTGGAGCATTTTCTCAATGAGCTCATCCTGTTCCAAAAGGGCCAGACCAACACC

CCACCACCCTTCGAGATCTTCTTCTGCTTTGGGGAAGAATGGCCTGACCGCAAACCCCGA

GAGAAGAAGCTCATTACTGTACAGGTGGTGCCTGTAGCAGCTCGACTGCTGCTGGAGATG

TTCTCAGGGGAGCTATCTTGGTCAGCTGATAGTATCCGGCTACAGATCTCAAACCCAGAC

CTCAAAGACCGCATGGTGGAGCAATTCAAGGAGCTCCATCACATCTGGCAGTCCCAGCAG

CGGTTGCAGCCTGTGGCCCAGGCCCCTCCTGGAGCAGGCCTTGGTGTTGGCCAGGGGCCC

TGGCCTATGCACCCAGCTGGCATGCAATAACAAGGCTGCAGACGGTGACTGGCCCTGGCT

TCCTGGGTGGCGGTGCGGACTGATGTGGAGATGTGACAGCCCCGATGAGCACCTGGCTGG

CTGCAGGGTCCTACCTCTGGGTTTCCTGGAAGTGGATTTGGGCCAAGAAGGAGAGGGAGA

AAGGCCCGAGCCCCTGCCTTCCCGGGCCTTTCTCTCCTGGGCTGTCTCTGGTCTGGTCAG

CCTGGCTCTCGGGAAATTCAGCCATGAGCAGGGAAAGAACTCTCCCAACCCTGGGGCCTA

GCTGTATAGGAGGAATTGCCTAAGGGTGGCCCACTCTTGTGATTGCCCCATTTCCTCTGG

CAACAAAAGCCAGAGTGTTGTGGGCCAAGTCCCCCCACAGGGCCTCTGCAGGGCATGGCC

CTGATTTCCCTGGTTTGAGACTCACTTCCTCATCTCCCTGTCCTCTGAGATAATATGAGT

GAGCACTTAGGTATCATATCAGATGCTCAAGGCTGGCAGCTACCCCCTTCTTGAGAGTCC

AAGAACCTGGAGCAGAAATAATTTTTATGTATTTTTGGATTAATGAATGTTAAAAACAGA

CTCAGCTGTTTCTTTCCTTTTACTACTACCAGTTGCTCCCATGCTGCTCCACCAGGCCCT

GTTTCGGATGCCAACTGGCCCACTCCCCAAGCACTTGCCCCCAGCTTGCGACCATTGGCA

CTGGGAGGGCCTGGCTTCTGGGCTGATGGGTCAGTTGGGCCTTCATAAACACTCACCTGG

CTGGCTTTGCCTTCCAGGAGGAAGCTGGCTGAAGCAAGGGTGTGGAATTTTAAATGTGTG

CACAGTCTGGAAAACTGTCAGAATCAGTTTTCCCATAAAAGGGTGGGCTAGCATTGCAGC

TGCATTTGGGACCATTCAAATCTGTCACTCTCTTGTGTATATTCCTGTGCTATTAAATAT

ATCAGGGCAGTGCATGTAAATCATCCTGATATATTTAATATATTTATTATATTGTCCCCC

GAGGTGGGGACAGTGAGTGAGTTCTCTTAGTCCCCCCAGAGCTGGTTGTTAAAGAGCCTG

GCACCTACCCGCTCTCACTTCATCTGTGTCATCTCTGCACACTCCAGCCCACTTTCTGCC

TTCAGCCATTGAGTGGAAGCTGCCCCAGGCCCTTACCAGGTGCAGATGCCCAATCTTGAT

GCCCAGCCATCAGAACTGTGAGCCAAATAAACCTTTTTCTGTATAAA

>NV6

GCCCGGCAGGTTGGCGGACCGGCGGGAGGCGCAGCCTGGGCAGAGCTCAGCTTGGTCCCG

CCGCCCGGCCGGTGCTCCCTGGCGCAGCCACGCAGGCGCACCGCAGACAGACCCCTCTGC

CATGAACCAGTCCATCCCAGTGGCTCCCACCCCACCCCGCCGCGTGCGGCTGAAGCCCTG

GCTGGTGGCCCAGGTGAACACACGAACCAGCTGCTGGATGTCCTGGACCGCGGGCTCATC

CTCCAGCTACAGGGCCAGGACCTTTATGCCATCCGCCTGTGTCAGTGCAAGGTGTTCTGG

AGCGGGCCTTGTGCCTCAGCCCATGACTCATGCCCCAACCCCATCCAGCGGGAGGTCAAG

ACCAAGCTTTTCAGCCTGGAGCATTTTCTCAATGAGCTCATCCTGTTCCAAAAGGGCCAG

ACCAACACCCCACCACCCTTCGAGATCTTCTTCTGCTTTGGGGAAGAATGGCCTGACCGC

AAACCCCGAGAGAAGAAGCTCATTACTGTACAGGTGGTGCCTGTAGCAGCTCGACTGCTG

CTGGAGATGTTCTCAGGGGAGCTATCTTGGTCAGCTGATAGTATCCGGCTACAGATCTCA

AACCCAGACCTCAAAGACCGCATGGTGGAGCAATTCAAGGAGCTCCATCACATCTGGCAG

TCCCAGCAGCGGTTGCAGCCTGTGGCCCAGGCCCCTCCTGGAGCAGGCCTTGGTGTTGGC

CAGGGGCCCTGGCCTATGCACCCAGCTGGCATGCAATAACAAGGCTGCAGACGGTGACTG

GCCCTGGCTTCCTGGGTGGCGGTGCGGACTGATGTGGAGATGTGACAGCCCCGATGAGCA

CCTGGCTGGCTGCAGGGTCCTACCTCTGGGTTTCCTGGAAGTGGATTTGGGCCAAGAAGG

AGAGGGAGAAAGGCCCGAGCCCCTGCCTTCCCGGGCCTTTCTCTCCTGGGCTGTCTCTGG

TCTGGTCAGCCTGGCTCTCGGGAAATTCAGCCATGAGCAGGGAAAGAACTCTCCCAACCC

TGGGGCCTAGCTGTATAGGAGGAATTGCCTAAGGGTGGCCCACTCTTGTGATTGCCCCAT

TTCCTCTGGCAACAAAAGCCAGAGTGTTGTGGGCCAAGTCCCCCCACAGGGCCTCTGCAG

GGCATGGCCCTGATTTCCCTGGTTTGAGACTCACTTCCTCATCTCCCTGTCCTCTGAGAT

AATATGAGTGAGCACTTAGGTATCATATCAGATGCTCAAGGCTGGCAGCTACCCCCTTCT

TGAGAGTCCAAGAACCTGGAGCAGAAATAATTTTTATGTATTTTTGGATTAATGAATGTT

AAAAACAGACTCAGCTGTTTCTTTCCTTTTACTACTACCAGTTGCTCCCATGCTGCTCCA

CCAGGCCCTGTTTCGGATGCCAACTGGCCCACTCCCCAAGCACTTGCCCCCAGCTTGCGA

CCATTGGCACTGGGAGGGCCTGGCTTCTGGGCTGATGGGTCAGTTGGGCCTTCATAAACA

CTCACCTGGCTGGCTTTGCCTTCCAGGAGGAAGCTGGCTGAAGCAAGGGTGTGGAATTTT

AAATGTGTGCACAGTCTGGAAAACTGTCAGAATCAGTTTTCCCATAAAAGGGTGGGCTAG

CATTGCAGCTGCATTTGGGACCATTCAAATCTGTCACTCTCTTGTGTATATTCCTGTGCT

ATTAAATATATCAGGGCAGTGCATGTAAATCATCCTGATATATTTAATATATTTATTATA

TTGTCCCCCGAGGTGGGGACAGTGAGTGAGTTCTCTTAGTCCCCCCAGAGCTGGTTGTTA

AAGAGCCTGGCACCTACCCGCTCTCACTTCATCTGTGTCATCTCTGCACACTCCAGCCCA

CTTTCTGCCTTCAGCCATTGAGTGGAAGCTGCCCCAGGCCCTTACCAGGTGCAGATGCCC

AATCTTGATGCCCAGCCATCAGAACTGTGAGCCAAATAAACCTTTTTCTGTATAAA

>NV7

GCCCGGCAGGTTGGCGGACCGGCGGGAGGCGCAGCCTGGGCAGAGCTCAGCTTGGTCCCG

CCGCCCGGCCGGTGCTCCCTGGCGCAGCCACGCAGGCGCACCGCAGACAGACCCCTCTGC

CATGAACCAGTCCATCCCAGTGGCTCCCACCCCACCCCGCCGCGTGCGGCTGAAGCCCTG

GCTGGTGGCCCAGGTGAACAGCTGCCAGTACCCAGGGCTTCAATGGGTCAACGGGGAAAA

GAAATTATTCTGCATCCCCTGGAGGCATGCCACAAGGCATGGTCCCAGCCAGGACGGAGA

TAACACCATCTTCAAGGCCTGGGCCAAGGAGACAGGGAAATACACCGAAGGCGTGGATGA

AGCCGATCCGGCCAAGTGGAAGGCCAACCTGCGCTGTGCCCTTAACAAGAGCCGGGACTT

CCGCCTCATCTACGACGGGCCCCGGGACATGCCACCTCAGCCCTACAAGATCTACGAGAC

TCCCAGCCCCCTGAGGATTACTCTTTTGGTGCAGGAGAGGAGGAGGAAGAAGAGGAAGAG

AGGATGTCAAGTGGCCGCCCACTCTGCAGCCGCCCACTCTGCAGCCGCCCGTGGTGCTGG

GTCCCCCTGCTCCAGACCCCAGCCCCCTGGCTCCTCCCCCTGGCAACCCTGCTGGCTTCA

GGGAGCTTCTCTCTGAGGTCCTGGAGCCTGGGCCCCTGCCTGCCAGCCTGCCCCCTGCAG

GCGAACAGCTCCTGCCAGACCTGCTGATCAGCCCCCACATGCTGCCTCTGACCGACCTGG

AGATCAAGTTTCAGTACCGGGGGCGGCCACCCCGGGCCCTCACCATCAGCAACCCCCATG

GCTGCCGGCTCTTCTACAGCCAGCTGGAGGCCACCCAGGAGCAGGTGGAACTCTTCGGCC

CCATAAGCCTGGAGCAAGTGCGCTTCCCCAGCCCTGAGGACATCCCCAGTGACAAGCAGC

GCTTCTACACGAACCAGCTGCTGGATGTCCTGGACCGCGGGCTCATCCTCCAGCTACAGG

GCCAGGACCTTTATGCCATCCGCCTGTGTCAGTGCAAGGTGTTCTGGAGCGGGCCTTGTG

CCTCAGCCCATGACTCATGCCCCAACCCCATCCAGCGGGAGGTCAAGACCAAGCTTTTCA

GCCTGGAGCATTTTCTCAATGAGCTCATCCTGTTCCAAAAGGGCCAGACCAACACCCCAC

CACCCTTCGAGATCTTCTTCTGCTTTGGGGAAGAATGGCCTGACCGCAAACCCCGAGAGA

AGAAGCTCATTACTGTACAGGTGGTGCCTGTAGCAGCTCGACTGCTGCTGGAGATGTTCT

CAGGGGAGCTATCTTGGTCAGCTGATAGTATCCGGCTACAGATCTCAAACCCAGACCTCA

AAGACCGCATGGTGGAGCAATTCAAGGAGCTCCATCACATCTGGCAGTCCCAGCAGCGGT

TGCAGCCTGTGGCCCAGGCCCCTCCTGGAGCAGGCCTTGGTGTTGGCCAGGGGCCCTGGC

CTATGCACCCAGCTGGCATGCAATAACAAGGCTGCAGACGGTGACTGGCCCTGGCTTCCT

GGGTGGCGGTGCGGACTGATGTGGAGATGTGACAGCCCCGATGAGCACCTGGCTGGCTGC

AGGGTCCTACCTCTGGGTTTCCTGGAAGTGGATTTGGGCCAAGAAGGAGAGGGAGAAAGG

CCCGAGCCCCTGCCTTCCCGGGCCTTTCTCTCCTGGGCTGTCTCTGGTCTGGTCAGCCTG

GCTCTCGGGAAATTCAGCCATGAGCAGGGAAAGAACTCTCCCAACCCTGGGGCCTAGCTG

TATAGGAGGAATTGCCTAAGGGTGGCCCACTCTTGTGATTGCCCCATTTCCTCTGGCAAC

AAAAGCCAGAGTGTTGTGGGCCAAGTCCCCCCACAGGGCCTCTGCAGGGCATGGCCCTGA

TTTCCCTGGTTTGAGACTCACTTCCTCATCTCCCTGTCCTCTGAGATAATATGAGTGAGC

ACTTAGGTATCATATCAGATGCTCAAGGCTGGCAGCTACCCCCTTCTTGAGAGTCCAAGA

ACCTGGAGCAGAAATAATTTTTATGTATTTTTGGATTAATGAATGTTAAAAACAGACTCA

GCTGTTTCTTTCCTTTTACTACTACCAGTTGCTCCCATGCTGCTCCACCAGGCCCTGTTT

CGGATGCCAACTGGCCCACTCCCCAAGCACTTGCCCCCAGCTTGCGACCATTGGCACTGG

GAGGGCCTGGCTTCTGGGCTGATGGGTCAGTTGGGCCTTCATAAACACTCACCTGGCTGG

CTTTGCCTTCCAGGAGGAAGCTGGCTGAAGCAAGGGTGTGGAATTTTAAATGTGTGCACA

GTCTGGAAAACTGTCAGAATCAGTTTTCCCATAAAAGGGTGGGCTAGCATTGCAGCTGCA

TTTGGGACCATTCAAATCTGTCACTCTCTTGTGTATATTCCTGTGCTATTAAATATATCA

GGGCAGTGCATGTAAATCATCCTGATATATTTAATATATTTATTATATTGTCCCCCGAGG

TGGGGACAGTGAGTGAGTTCTCTTAGTCCCCCCAGAGCTGGTTGTTAAAGAGCCTGGCAC

CTACCCGCTCTCACTTCATCTGTGTCATCTCTGCACACTCCAGCCCACTTTCTGCCTTCA

GCCATTGAGTGGAAGCTGCCCCAGGCCCTTACCAGGTGCAGATGCCCAATCTTGATGCCC

AGCCATCAGAACTGTGAGCCAAATAAACCTTTTTCTGTATAAA

>NV8

GCCCGGCAGGTTGGCGGACCGGCGGGAGGCGCAGCCTGGGCAGAGCTCAGCTTGGTCCCG

CCGCCCGGCCGGTGCTCCCTGGCGCAGCCACGCAGGCGCACCGCAGACAGACCCCTCTGC

CATGAACCAGTCCATCCCAGTGGCTCCCACCCCACCCCGCCGCGTGCGGCTGAAGCCCTG

GCTGGTGGCCCAGGTGAACAGCTGCCAGTACCCAGGGCTTCAATGGGTCAACGGGGAAAA

GAAATTATTCTGCATCCCCTGGAGGCATGCCACAAGGCATGGTCCCAGCCAGGACGGAGA

TAACACCATCTTCAAGGCCTGGGCCAAGGAGACAGGGAAATACACCGAAGGCGTGGATGA

AGCCGATCCGGCCAAGTGGAAGGCCAACCTGCGCTGTGCCCTTAACAAGAGCCGGGACTT

CCGCCTCATCTACGACGGGCCCCGGGACATGCCACCTCAGCCCTACAAGATCTACGAGGT

CTGCTCCAATGGCCCTGCTCCCACAGACTCCCAGCCCCCTGAGGATTACTCTTTTGGTGC

AGGAGAGGAGGAGGAAGAAGAGGAAGAGCTGCAGAGGATGTTGCCAAGCCTGAGCCTCAC

AGAGGATGTCAAGTGGCCGCCCACTCTGCAGCCGCCCACTCTGCGGCCGCCTACTCTGCA

GCCGCCCACTCTGCAGCCGCCCGTGGTGCTGGGTCCCCCTGCTCCAGACCCCAGCCCCCT

GGCTCCTCCCCCTGGCAACCCTGCTGGCTTCAGGGAGCTTCTCTCTGAGGTCCTGGAGCC

TGGGCCCCTGCCTGCCAGCCTGCCCCCTGCAGGCGAACAGCTCCTGCCAGACCTGCTGAT

CAGCCCCCACATGCTGCCTCTGACCGACCTGGAGATCAAGTTTCAGTACCGGGGGCGGCC

ACCCCGGGCCCTCACCATCAGCAACCCCCATGGCTGCCGGCTCTTCTACAGCCAGCTGGA

GGCCACCCAGGAGCAGGTGGAACTCTTCGGCCCCATAAGCCTGGAGCAAGTGCGCTTCCC

CAGCCCTGAGGACATCCCCAGTGACAAGCAGCGCTTCTACACGAACCAGCTGCTGGATGT

CCTGGACCGCGGGCTCATCCTCCAGCTACAGGGCCAGGACCTTTATGCCATCCGCCTGTG

TCAGTGCAAGGTGTTCTGGAGCGGGCCTTGTGCCTCAGCCCATGACTCATGCCCCAACCC

CATCCAGCGGGAGGTCAAGACCAAGCTTTTCAGCCTGGAGCATTTTCTCAATGGGGCCAG

ACCAACACCCCACCACCCTTCGAGATCTTCTTCTGCTTTGGGGAAGAATGGCCTGACCGC

AAACCCCGAGAGAAGAAGCTCATTACTGTACAGGTGGTGCCTGTAGCAGCTCGACTGCTG

CTGGAGATGTTCTCAGGGGAGCTATCTTGGTCAGCTGATAGTATCCGGCTACAGATCTCA

AACCCAGACCTCAAAGACCGCATGGTGGAGCAATTCAAGGAGCTCCATCACATCTGGCAG

TCCCAGCAGCGGTTGCAGCCTGTGGCCCAGGCCCCTCCTGGAGCAGGCCTTGGTGTTGGC

CAGGGGCCCTGGCCTATGCACCCAGCTGGCATGCAATAACAAGGCTGCAGACGGTGACTG

GCCCTGGCTTCCTGGGTGGCGGTGCGGACTGATGTGGAGATGTGACAGCCCCGATGAGCA

CCTGGCTGGCTGCAGGGTCCTACCTCTGGGTTTCCTGGAAGTGGATTTGGGCCAAGAAGG

AGAGGGAGAAAGGCCCGAGCCCCTGCCTTCCCGGGCCTTTCTCTCCTGGGCTGTCTCTGG

TCTGGTCAGCCTGGCTCTCGGGAAATTCAGCCATGAGCAGGGAAAGAACTCTCCCAACCC

TGGGGCCTAGCTGTATAGGAGGAATTGCCTAAGGGTGGCCCACTCTTGTGATTGCCCCAT

TTCCTCTGGCAACAAAAGCCAGAGTGTTGTGGGCCAAGTCCCCCCACAGGGCCTCTGCAG

GGCATGGCCCTGATTTCCCTGGTTTGAGACTCACTTCCTCATCTCCCTGTCCTCTGAGAT

AATATGAGTGAGCACTTAGGTATCATATCAGATGCTCAAGGCTGGCAGCTACCCCCTTCT

TGAGAGTCCAAGAACCTGGAGCAGAAATAATTTTTATGTATTTTTGGATTAATGAATGTT

AAAAACAGACTCAGCTGTTTCTTTCCTTTTACTACTACCAGTTGCTCCCATGCTGCTCCA

CCAGGCCCTGTTTCGGATGCCAACTGGCCCACTCCCCAAGCACTTGCCCCCAGCTTGCGA

CCATTGGCACTGGGAGGGCCTGGCTTCTGGGCTGATGGGTCAGTTGGGCCTTCATAAACA

CTCACCTGGCTGGCTTTGCCTTCCAGGAGGAAGCTGGCTGAAGCAAGGGTGTGGAATTTT

AAATGTGTGCACAGTCTGGAAAACTGTCAGAATCAGTTTTCCCATAAAAGGGTGGGCTAG

CATTGCAGCTGCATTTGGGACCATTCAAATCTGTCACTCTCTTGTGTATATTCCTGTGCT

ATTAAATATATCAGGGCAGTGCATGTAAATCATCCTGATATATTTAATATATTTATTATA

TTGTCCCCCGAGGTGGGGACAGTGAGTGAGTTCTCTTAGTCCCCCCAGAGCTGGTTGTTA

AAGAGCCTGGCACCTACCCGCTCTCACTTCATCTGTGTCATCTCTGCACACTCCAGCCCA

CTTTCTGCCTTCAGCCATTGAGTGGAAGCTGCCCCAGGCCCTTACCAGGTGCAGATGCCC

AATCTTGATGCCCAGCCATCAGAACTGTGAGCCAAATAAACCTTTTTCTGTATAAA

>NV9

GCCCGGCAGGTTGGCGGACCGGCGGGAGGCGCAGCCTGGGCAGAGCTCAGCTTGGTCCCG

CCGCCCGGCCGGTGCTCCCTGGCGCAGCCACGCAGGCGCACCGCAGACAGACCCCTCTGC

CATGAACCAGTCCATCCCAGTGGCTCCCACCCCACCCCGCCGCGTGCGGCTGAAGCCCTG

GCTGGTGGCCCAGGTGAACAGCTGCCAGTACCCAGGGCTTCAATGGGTCAACGGGGAAAA

GAAATTATTCTGCATCCCCTGGAGGCATGCCACAAGGCATGGTCCCAGCCAGGACGGAGA

TAACACCATCTTCAAGGCCTGGGCCAAGGAGACAGGGAAATACACCGAAGGCGTGGATGA

AGCCGATCCGGCCAAGTGGAAGGCCAACCTGCGCTGTGCCCTTAACAAGAGCCGGGACTT

CCGCCTCATCTACGACGGGCCCCGGGACATGCCACCTCAGCCCTACAAGATCTACGAGGT

CTGCTCCAATGGCCCTGCTCCCACAGACTCCCAGCCCCCTGAGGATTACTCTTTTGGTGC

AGGAGAGGAGGAGGAAGAAGAGGAAGAGCTGCAGAGGATGTTGCCAAGCCTGAGCCTCAC

AGAGGATGTCAAGTGGCCGCCCACTCTGCAGCCGCCCACTCTGCGGCCGCCTACTCTGCA

GCCGCCCACTCTGCAGCCGCCCGTGGTGCTGGGTCCCCCTGCTCCAGACCCCAGCCCCCT

GGCTCCTCCCCCTGGCAACCCTGCTGGCTTCAGGGAGCTTCTCTCTGAGGTCCTGGAGCC

TGGGCCCCTGCCTGCCAGCCTGCCCCCTGCAGGCGAACAGCTCCTGCCAGACCTGCTGAT

CAGCCCCCACATGCTGCCTCTGACCGACCTGGAGATCAAGTTTCAGTACCGGGGGCGGCC

ACCCCGGGCCCTCACCATCAGCAACCCCCATGGCTGCCGGCTCTTCTACAGCCAGCTGGA

GGCCACCCAGGAGCAGGTGGAACTCTTCGGCCCCATAAGCCTGGAGCAAGTGCGCTTCCC

CAGCCCTGAGGACATCCCCAGTGACAAGCAGCGCTTCTACACGAACCAGCTGCTGGATGT

CCTGGACCGCGGGCTCATCCTCCAGCTACAGGGCCAGGACCTTTATGCCATCCGCCTGTG

TCAGTGCAAGAGCTCATCCTGTTCCAAAAGGGCCAGACCAACACCCCACCACCCTTCGAG

ATCTTCTTCTGCTTTGGGGAAGAATGGCCTGACCGCAAACCCCGAGAGAAGAAGCTCATT

ACTGTACAGGTGGTGCCTGTAGCAGCTCGACTGCTGCTGGAGATGTTCTCAGGGGAGCTA

TCTTGGTCAGCTGATAGTATCCGGCTACAGATCTCAAACCCAGACCTCAAAGACCGCATG

GTGGAGCAATTCAAGGAGCTCCATCACATCTGGCAGTCCCAGCAGCGGTTGCAGCCTGTG

GCCCAGGCCCCTCCTGGAGCAGGCCTTGGTGTTGGCCAGGGGCCCTGGCCTATGCACCCA

GCTGGCATGCAATAACAAGGCTGCAGACGGTGACTGGCCCTGGCTTCCTGGGTGGCGGTG

CGGACTGATGTGGAGATGTGACAGCCCCGATGAGCACCTGGCTGGCTGCAGGGTCCTACC

TCTGGGTTTCCTGGAAGTGGATTTGGGCCAAGAAGGAGAGGGAGAAAGGCCCGAGCCCCT

GCCTTCCCGGGCCTTTCTCTCCTGGGCTGTCTCTGGTCTGGTCAGCCTGGCTCTCGGGAA

ATTCAGCCATGAGCAGGGAAAGAACTCTCCCAACCCTGGGGCCTAGCTGTATAGGAGGAA

TTGCCTAAGGGTGGCCCACTCTTGTGATTGCCCCATTTCCTCTGGCAACAAAAGCCAGAG

TGTTGTGGGCCAAGTCCCCCCACAGGGCCTCTGCAGGGCATGGCCCTGATTTCCCTGGTT

TGAGACTCACTTCCTCATCTCCCTGTCCTCTGAGATAATATGAGTGAGCACTTAGGTATC

ATATCAGATGCTCAAGGCTGGCAGCTACCCCCTTCTTGAGAGTCCAAGAACCTGGAGCAG

AAATAATTTTTATGTATTTTTGGATTAATGAATGTTAAAAACAGACTCAGCTGTTTCTTT

CCTTTTACTACTACCAGTTGCTCCCATGCTGCTCCACCAGGCCCTGTTTCGGATGCCAAC

TGGCCCACTCCCCAAGCACTTGCCCCCAGCTTGCGACCATTGGCACTGGGAGGGCCTGGC

TTCTGGGCTGATGGGTCAGTTGGGCCTTCATAAACACTCACCTGGCTGGCTTTGCCTTCC

AGGAGGAAGCTGGCTGAAGCAAGGGTGTGGAATTTTAAATGTGTGCACAGTCTGGAAAAC

TGTCAGAATCAGTTTTCCCATAAAAGGGTGGGCTAGCATTGCAGCTGCATTTGGGACCAT

TCAAATCTGTCACTCTCTTGTGTATATTCCTGTGCTATTAAATATATCAGGGCAGTGCAT

GTAAATCATCCTGATATATTTAATATATTTATTATATTGTCCCCCGAGGTGGGGACAGTG

AGTGAGTTCTCTTAGTCCCCCCAGAGCTGGTTGTTAAAGAGCCTGGCACCTACCCGCTCT

CACTTCATCTGTGTCATCTCTGCACACTCCAGCCCACTTTCTGCCTTCAGCCATTGAGTG

GAAGCTGCCCCAGGCCCTTACCAGGTGCAGATGCCCAATCTTGATGCCCAGCCATCAGAA

CTGTGAGCCAAATAAACCTTTTTCTGTATAAA

>NV10

GCCCGGCAGGTTGGCGGACCGGCGGGAGGCGCAGCCTGGGCAGAGCTCAGCTTGGTCCCG

CCGCCCGGCCGGTGCTCCCTGGCGCAGCCACGCAGGCGCACCGCAGACAGACCCCTCTGC

CATGAACCAGTCCATCCCAGTGGCTCCCACCCCACCCCGCCGCGTGCGGCTGAAGCCCTG

GCTGGTGGCCCAGGTGAACAGCTGCCAGTACCCAGGGCTTCAATGGGTCAACGGGGAAAA

GAAATTATTCTGCATCCCCTGGAGGCATGCCACAAGGCATGGTCCCAGCCAGGACGGAGA

TAACACCATCTTCAAGGCCTGGGCCAAGGAGACAGGGAAATACACCGAAGGCGTGGATGA

AGCCGATCCGGCCAAGTGGAAGGCCAACCTGCGCTGTGCCCTTAACAAGAGCCGGGACTT

CCGCCTCATCTACGACGGGCCCCGGGACATGCCACCTCAGCCCTACAAGATCTACGAGGT

CTGCTCCAATGGCCCTGCTCCCACAGACTCCCAGCCCCCTGAGGATTACTCTTTTGGTGC

AGGAGAGGAGGAGGAAGAAGAGGAAGAGCTGCAGAGGATGTTGCCAAGCCTGAGCCTCAC

AGAGGATGTCAAGTGGCCGCCCACTCTGCAGCCGCCCACTCTGCGGCCGCCTACTCTGCA

GCCGCCCACTCTGCAGCCGCCCGTGGTGCTGGGTCCCCCTGCTCCAGACCCCAGCCCCCT

GGCTCCTCCCCCTGGCAACCCTGCTGGCTTCAGGGAGCTTCTCTCTGAGGTCCTGGAGCC

TGGGCCCCTGCCTGCCAGCCTGCCCCCTGCAGGCGAACAGCTCCTGCCAGACCTGCTGAT

CAGCCCCCACATGCTGCCTCGTAAGGACCCATGGCTGGGCACGGGGAAGCAGTGCTGGGG

GATTGGGTGACCGACCTGGAGATCAAGTTTCAGTACCGGGGGCGGCCACCCCGGGCCCTC

ACCATCAGCAACCCCCATGGCTGCCGGCTCTTCTACAGCCAGCTGGAGGCCACCCAGGAG

CAGGTGGAACTCTTCGGCCCCATAAGCCTGGAGCAAGTGCGCTTCCCCAGCCCTGAGGAC

ATCCCCAGTGACAAGCAGCGCTTCTACACGAACCAGCTGCTGGATGTCCTGGACCGCGGG

CTCATCCTCCAGCTACAGGGCCAGGACCTTTATGCCATCCGCCTGTGTCAGTGCAAGGTG

TTCTGGAGCGGGCCTTGTGCCTCAGCCCATGACTCATGCCCCAACCCCATCCAGCGGGAG

GTCAAGACCAAGCTTTTCAGCCTGGAGCATTTTCTCAATGAGCTCATCCTGTTCCAAAAG

GGCCAGACCAACACCCCACCACCCTTCGAGATCTTCTTCTGCTTTGGGGAAGAATGGCCT

GACCGCAAACCCCGAGAGAAGAAGCTCATTACTGTACAGGTGGTGCCTGTAGCAGCTCGA

CTGCTGCTGGAGATGTTCTCAGGGGAGCTATCTTGGTCAGCTGATAGTATCCGGCTACAG

ATCTCAAACCCAGACCTCAAAGACCGCATGGTGGAGCAATTCAAGGAGCTCCATCACATC

TGGCAGTCCCAGCAGCGGTTGCAGCCTGTGGCCCAGGCCCCTCCTGGAGCAGGCCTTGGT

GTTGGCCAGGGGCCCTGGCCTATGCACCCAGCTGGCATGCAATAACAAGGCTGCAGACGG

TGACTGGCCCTGGCTTCCTGGGTGGCGGTGCGGACTGATGTGGAGATGTGACAGCCCCGA

TGAGCACCTGGCTGGCTGCAGGGTCCTACCTCTGGGTTTCCTGGAAGTGGATTTGGGCCA

AGAAGGAGAGGGAGAAAGGCCCGAGCCCCTGCCTTCCCGGGCCTTTCTCTCCTGGGCTGT

CTCTGGTCTGGTCAGCCTGGCTCTCGGGAAATTCAGCCATGAGCAGGGAAAGAACTCTCC

CAACCCTGGGGCCTAGCTGTATAGGAGGAATTGCCTAAGGGTGGCCCACTCTTGTGATTG

CCCCATTTCCTCTGGCAACAAAAGCCAGAGTGTTGTGGGCCAAGTCCCCCCACAGGGCCT

CTGCAGGGCATGGCCCTGATTTCCCTGGTTTGAGACTCACTTCCTCATCTCCCTGTCCTC

TGAGATAATATGAGTGAGCACTTAGGTATCATATCAGATGCTCAAGGCTGGCAGCTACCC

CCTTCTTGAGAGTCCAAGAACCTGGAGCAGAAATAATTTTTATGTATTTTTGGATTAATG

AATGTTAAAAACAGACTCAGCTGTTTCTTTCCTTTTACTACTACCAGTTGCTCCCATGCT

GCTCCACCAGGCCCTGTTTCGGATGCCAACTGGCCCACTCCCCAAGCACTTGCCCCCAGC

TTGCGACCATTGGCACTGGGAGGGCCTGGCTTCTGGGCTGATGGGTCAGTTGGGCCTTCA

TAAACACTCACCTGGCTGGCTTTGCCTTCCAGGAGGAAGCTGGCTGAAGCAAGGGTGTGG

AATTTTAAATGTGTGCACAGTCTGGAAAACTGTCAGAATCAGTTTTCCCATAAAAGGGTG

GGCTAGCATTGCAGCTGCATTTGGGACCATTCAAATCTGTCACTCTCTTGTGTATATTCC

TGTGCTATTAAATATATCAGGGCAGTGCATGTAAATCATCCTGATATATTTAATATATTT

ATTATATTGTCCCCCGAGGTGGGGACAGTGAGTGAGTTCTCTTAGTCCCCCCAGAGCTGG

TTGTTAAAGAGCCTGGCACCTACCCGCTCTCACTTCATCTGTGTCATCTCTGCACACTCC

AGCCCACTTTCTGCCTTCAGCCATTGAGTGGAAGCTGCCCCAGGCCCTTACCAGGTGCAG

ATGCCCAATCTTGATGCCCAGCCATCAGAACTGTGAGCCAAATAAACCTTTTTCTGTATA

AA

>NV11

GCCCGGCAGGTTGGCGGACCGGCGGGAGGCGCAGCCTGGGCAGAGCTCAGCTTGGTCCCG

CCGCCCGGCCGGTGCTCCCTGGCGCAGCCACGCAGGCGCACCGCAGACAGACCCCTCTGC

CATGAACCAGTCCATCCCAGTGGCTCCCACCCCACCCCGCCGCGTGCGGCTGAAGCCCTG

GCTGGTGGCCCAGGTGAACAGCTGCCAGTACCCAGGGCTTCAATGGGTCAACGGGGAAAA

GAAATTATTCTGCATCCCCTGGAGGCATGCCACAAGGCATGGTCCCAGCCAGGACGGAGA

TAACACCATCTTCAAGGCCTGGGCCAAGGAGACAGGGAAATACACCGAAGGCGTGGATGA

AGCCGATCCGGCCAAGTGGAAGGCCAACCTGCGCTGTGCCCTTAACAAGAGCCGGGACTT

CCGCCTCATCTACGACGGGCCCCGGGACATGCCACCTCAGCCCTACAAGATCTACGAGGT

CTGCTCCAATGGCCCTGCTCCCACAGACTCCCAGCCCCCTGAGGATTACTCTTTTGGTGC

AGGAGAGGAGGAGGAAGAAGAGGAAGAGATGCAGTGCAGTCTGGCCCCCACATGACACCC

TATTCTTTACTCAAAGAGGATGTCAAGTGGCCGCCCACTCTGCAGCCGCCCACTCTGCGG

CCGCCTACTCTGCAGCCGCCCACTCTGCAGCCGCCCGTGGTGCTGGGTCCCCCTGCTCCA

GACCCCAGCCCCCTGGCTCCTCCCCCTGGCAACCCTGCTGGCTTCAGGGAGCTTCTCTCT

GAGGTCCTGGAGCCTGGGCCCCTGCCTGCCAGCCTGCCCCCTGCAGGCGAACAGCTCCTG

CCAGACCTGCTGATCAGCCCCCACATGCTGCCTCTGACCGACCTGGAGATCAAGTTTCAG

TACCGGGGGCGGCCACCCCGGGCCCTCACCATCAGCAACCCCCATGGCTGCCGGCTCTTC

TACAGCCAGCTGGAGGCCACCCAGGAGCAGGTGGAACTCTTCGGCCCCATAAGCCTGGAG

CAAGTGCGCTTCCCCAGCCCTGAGGACATCCCCAGTGACAAGCAGCGCTTCTACACGAAC

CAGCTGCTGGATGTCCTGGACCGCGGGCTCATCCTCCAGCTACAGGGCCAGGACCTTTAT

GCCATCCGCCTGTGTCAGTGCAAGGTGTTCTGGAGCGGGCCTTGTGCCTCAGCCCATGAC

TCATGCCCCAACCCCATCCAGCGGGAGGTCAAGACCAAGCTTTTCAGCCTGGAGCATTTT

CTCAATGAGCTCATCCTGTTCCAAAAGGGCCAGACCAACACCCCACCACCCTTCGAGATC

TTCTTCTGCTTTGGGGAAGAATGGCCTGACCGCAAACCCCGAGAGAAGAAGCTCATTACT

GTACAGGTGGTGCCTGTAGCAGCTCGACTGCTGCTGGAGATGTTCTCAGGGGAGCTATCT

TGGTCAGCTGATAGTATCCGGCTACAGATCTCAAACCCAGACCTCAAAGACCGCATGGTG

GAGCAATTCAAGGAGCTCCATCACATCTGGCAGTCCCAGCAGCGGTTGCAGCCTGTGGCC

CAGGCCCCTCCTGGAGCAGGCCTTGGTGTTGGCCAGGGGCCCTGGCCTATGCACCCAGCT

GGCATGCAATAACAAGGCTGCAGACGGTGACTGGCCCTGGCTTCCTGGGTGGCGGTGCGG

ACTGATGTGGAGATGTGACAGCCCCGATGAGCACCTGGCTGGCTGCAGGGTCCTACCTCT

GGGTTTCCTGGAAGTGGATTTGGGCCAAGAAGGAGAGGGAGAAAGGCCCGAGCCCCTGCC

TTCCCGGGCCTTTCTCTCCTGGGCTGTCTCTGGTCTGGTCAGCCTGGCTCTCGGGAAATT

CAGCCATGAGCAGGGAAAGAACTCTCCCAACCCTGGGGCCTAGCTGTATAGGAGGAATTG

CCTAAGGGTGGCCCACTCTTGTGATTGCCCCATTTCCTCTGGCAACAAAAGCCAGAGTGT

TGTGGGCCAAGTCCCCCCACAGGGCCTCTGCAGGGCATGGCCCTGATTTCCCTGGTTTGA

GACTCACTTCCTCATCTCCCTGTCCTCTGAGATAATATGAGTGAGCACTTAGGTATCATA

TCAGATGCTCAAGGCTGGCAGCTACCCCCTTCTTGAGAGTCCAAGAACCTGGAGCAGAAA

TAATTTTTATGTATTTTTGGATTAATGAATGTTAAAAACAGACTCAGCTGTTTCTTTCCT

TTTACTACTACCAGTTGCTCCCATGCTGCTCCACCAGGCCCTGTTTCGGATGCCAACTGG

CCCACTCCCCAAGCACTTGCCCCCAGCTTGCGACCATTGGCACTGGGAGGGCCTGGCTTC

TGGGCTGATGGGTCAGTTGGGCCTTCATAAACACTCACCTGGCTGGCTTTGCCTTCCAGG

AGGAAGCTGGCTGAAGCAAGGGTGTGGAATTTTAAATGTGTGCACAGTCTGGAAAACTGT

CAGAATCAGTTTTCCCATAAAAGGGTGGGCTAGCATTGCAGCTGCATTTGGGACCATTCA

AATCTGTCACTCTCTTGTGTATATTCCTGTGCTATTAAATATATCAGGGCAGTGCATGTA

AATCATCCTGATATATTTAATATATTTATTATATTGTCCCCCGAGGTGGGGACAGTGAGT

GAGTTCTCTTAGTCCCCCCAGAGCTGGTTGTTAAAGAGCCTGGCACCTACCCGCTCTCAC

TTCATCTGTGTCATCTCTGCACACTCCAGCCCACTTTCTGCCTTCAGCCATTGAGTGGAA

GCTGCCCCAGGCCCTTACCAGGTGCAGATGCCCAATCTTGATGCCCAGCCATCAGAACTG

TGAGCCAAATAAACCTTTTTCTGTATAAA

>NV12

GCCCGGCAGGTTGGCGGACCGGCGGGAGGCGCAGCCTGGGCAGAGCTCAGCTTGGTCCCG

CCGCCCGGCCGGTGCTCCCTGGCGCAGCCACGCAGGCGCACCGCAGACAGACCCCTCTGC

CATGAACCAGTCCATCCCAGTGGCTCCCACCCCACCCCGCCGCGTGCGGCTGAAGCCCTG

GCTGGTGGCCCAGGTGAACAGCTGCCAGTACCCAGGGCTTCAATGGGTCAACGGGGAAAA

GAAATTATTCTGCATCCCCTGGAGGCATGCCACAAGGCATGGTCCCAGCCAGGACGGAGA

TAACACCATCTTCAAGGCCTGGGCCAAGGAGACAGGGAAATACACCGAAGGCGTGGATGA

AGCCGATCCGGCCAAGTGGAAGGCCAACCTGCGCTGTGCCCTTAACAAGAGCCGGGACTT

CCGCCTCATCTACGACGGGCCCCGGGACATGCCACCTCAGCCCTACAAGATCTACGAGGT

CTGCTCCAATGGCCCTGCTCCCACAGACTCCCAGCCCCCTGAGGATTACTCTTTTGGTGC

AGGAGAGGAGGAGGAAGAAGAGGAAGAGCTGCAGAGGATGTTGCCAAGCCTGAGCCTCAC

AGAGGATGTCAAGTGGCCGCCCACTCTGCAGCCGCCCACTCTGCGGCCGCCTACTCTGCA

GCCGCCCACTCTGCAGCCGCCCGTGGTGCTGGGTCCCCCTGCTCCAGACCCCAGCCCCCT

GGCTCCTCCCCCTGGCAACCCTGCTGGCTTCAGGGAGCTTCTCTCTGAGGTCCTGGAGCC

TGGGCCCCTGCCTGCCAGCCTGCCCCCTGCAGGCGAACAGCTCCTGCCAGACCTGCTGAT

CAGCCCCCACATGCTGCCTCTGACCGACCTGGAGATCAAGTTTCAGTACCGGGGGCGGCC

ACCCCGGGCCCTCACCATCAGCAACCCCCATGGCTGCCGGCTCTTCTACAGCCAGCTGGA

GGCCACCCAGGAGCAGGTGGAACTCTTCGGCCCCATAAGCCTGGAGCAAGTGCGCTTCCC

CAGCCCTGAGGACATCCCCAGTGACAAGCAGCGCTTCTACACGAACCAGCTGCTGGATGT

CCTGGACCGCGGGCTCATCCTCCAGCTACAGGGCCAGGACCTTTATGCCATCCGCCTGTG

TCAGTGCAAGGTGTTCTGGAGCGGGCCTTGTGCCTCAGCCCATGACTCATGCCCCAACCC

CATCCAGCGGGAGGTCAAGACCAAGCTTTTCAGCCTGGAGCATTTTCTCAATGAGCTCAT

CCTGTTCCAAAAGGGGTTCCAAAAGGGCCAGACCAACACCCCACCACCCTTCGAGATCTT

CTTCTGCTTTGGGGAAGAATGGCCTGACCGCAAACCCCGAGAGAAGAAGCTCATTACTGT

ACAGGTGGTGCCTGTAGCAGCTCGACTGCTGCTGGAGATGTTCTCAGGGGAGCTATCTTG

GTCAGCTGATAGTATCCGGCTACAGATCTCAAACCCAGACCTCAAAGACCGCATGGTGGA

GCAATTCAAGGAGCTCCATCACATCTGGCAGTCCCAGCAGCGGTTGCAGCCTGTGGCCCA

GGCCCCTCCTGGAGCAGGCCTTGGTGTTGGCCAGGGGCCCTGGCCTATGCACCCAGCTGG

CATGCAATAACAAGGCTGCAGACGGTGACTGGCCCTGGCTTCCTGGGTGGCGGTGCGGAC

TGATGTGGAGATGTGACAGCCCCGATGAGCACCTGGCTGGCTGCAGGGTCCTACCTCTGG

GTTTCCTGGAAGTGGATTTGGGCCAAGAAGGAGAGGGAGAAAGGCCCGAGCCCCTGCCTT

CCCGGGCCTTTCTCTCCTGGGCTGTCTCTGGTCTGGTCAGCCTGGCTCTCGGGAAATTCA

GCCATGAGCAGGGAAAGAACTCTCCCAACCCTGGGGCCTAGCTGTATAGGAGGAATTGCC

TAAGGGTGGCCCACTCTTGTGATTGCCCCATTTCCTCTGGCAACAAAAGCCAGAGTGTTG

TGGGCCAAGTCCCCCCACAGGGCCTCTGCAGGGCATGGCCCTGATTTCCCTGGTTTGAGA

CTCACTTCCTCATCTCCCTGTCCTCTGAGATAATATGAGTGAGCACTTAGGTATCATATC

AGATGCTCAAGGCTGGCAGCTACCCCCTTCTTGAGAGTCCAAGAACCTGGAGCAGAAATA

ATTTTTATGTATTTTTGGATTAATGAATGTTAAAAACAGACTCAGCTGTTTCTTTCCTTT

TACTACTACCAGTTGCTCCCATGCTGCTCCACCAGGCCCTGTTTCGGATGCCAACTGGCC

CACTCCCCAAGCACTTGCCCCCAGCTTGCGACCATTGGCACTGGGAGGGCCTGGCTTCTG

GGCTGATGGGTCAGTTGGGCCTTCATAAACACTCACCTGGCTGGCTTTGCCTTCCAGGAG

GAAGCTGGCTGAAGCAAGGGTGTGGAATTTTAAATGTGTGCACAGTCTGGAAAACTGTCA

GAATCAGTTTTCCCATAAAAGGGTGGGCTAGCATTGCAGCTGCATTTGGGACCATTCAAA

TCTGTCACTCTCTTGTGTATATTCCTGTGCTATTAAATATATCAGGGCAGTGCATGTAAA

TCATCCTGATATATTTAATATATTTATTATATTGTCCCCCGAGGTGGGGACAGTGAGTGA

GTTCTCTTAGTCCCCCCAGAGCTGGTTGTTAAAGAGCCTGGCACCTACCCGCTCTCACTT

CATCTGTGTCATCTCTGCACACTCCAGCCCACTTTCTGCCTTCAGCCATTGAGTGGAAGC

TGCCCCAGGCCCTTACCAGGTGCAGATGCCCAATCTTGATGCCCAGCCATCAGAACTGTG

AGCCAAATAAACCTTTTTCTGTATAAA

>NV13

GCCCGGCAGGTTGGCGGACCGGCGGGAGGCGCAGCCTGGGCAGAGCTCAGCTTGGTCCCG

CCGCCCGGCCGGTGCTCCCTGGCGCAGCCACGCAGGCGCACCGCAGACAGACCCCTCTGC

CATGAACCAGTCCATCCCAGTGGCTCCCACCCCACCCCGCCGCGTGCGGCTGAAGCCCTG

GCTGGTGGCCCAGGTGAACAGCTGCCAGTACCCAGGGCTTCAATGGGTCAACGGGGAAAA

GAAATTATTCTGCATCCCCTGGAGGCATGCCACAAGGCATGGTCCCAGCCAGGACGGAGA

TAACACCATCTTCAAGGCCTGGGCCAAGGAGACAGGGAAATACACCGAAGGCGTGGATGA

AGCCGATCCGGCCAAGTGGAAGGCCAACCTGCGCTGTGCCCTTAACAAGAGCCGGGACTT

CCGCCTCATCTACGACGGGCCCCGGGACATGCCACCTCAGCCCTACAAGATCTACGAGAC

TCCCAGCCCCCTGAGGATTACTCTTTTGGTGCAGGAGAGGAGGAGGAAGAAGAGGAAGAG

CTGCAGAGGATGTTGCCAAGCCTGAGCCTCACAGCCGCCCACTCTGCAGCCGCCCGTGGT

GCTGGGTCCCCCTGCTCCAGACCCCAGCCCCCTGGCTCCTCCCCCTGGCAACCCTGCTGG

CTTCAGGGAGCTTCTCTCTGAGGTCCTGGAGCCTGGGCCCCTGCCTGCCAGCCTGCCCCC

TGCAGGCGAACAGCTCCTGCCAGACCTGCTGATCAGCCCCCACATGCTGCCTCTGACCGA

CCTGGAGATCAAGTTTCAGTACCGGGGGCGGCCACCCCGGGCCCTCACCATCAGCAACCC

CCATGGCTGCCGGCTCTTCTACAGCCAGCTGGAGGCCACCCAGGAGCAGGTGGAACTCTT

CGGCCCCATAAGCCTGGAGCAAGTGCGCTTCCCCAGCCCTGAGGACATCCCCAGTGACAA

GCAGCGCTTCTACACGAACCAGCTGCTGGATGTCCTGGACCGCGGGCTCATCCTCCAGCT

ACAGGGCCAGGACCTTTATGCCATCCGCCTGTGTCAGTGCAAGGTGTTCTGGAGCGGGCC

TTGTGCCTCAGCCCATGACTCATGCCCCAACCCCATCCAGCGGGAGGTCAAGACCAAGCT

TTTCAGCCTGGAGCATTTTCTCAATGAGCTCATCCTGTTCCAAAAGGGCCAGACCAACAC

CCCACCACCCTTCGAGATCTTCTTCTGCTTTGGGGAAGAATGGCCTGACCGCAAACCCCG

AGAGAAGAAGCTCATTACTGTACAGGTGGTGCCTGTAGCAGCTCGACTGCTGCTGGAGAT

GTTCTCAGGGGAGCTATCTTGGTCAGCTGATAGTATCCGGCTACAGATCTCAAACCCAGA

CCTCAAAGACCGCATGGTGGAGCAATTCAAGGAGCTCCATCACATCTGGCAGTCCCAGCA

GCGGTTGCAGCCTGTGGCCCAGGCCCCTCCTGGAGCAGGCCTTGGTGTTGGCCAGGGGCC

CTGGCCTATGCACCCAGCTGGCATGCAATAACAAGGCTGCAGACGGTGACTGGCCCTGGC

TTCCTGGGTGGCGGTGCGGACTGATGTGGAGATGTGACAGCCCCGATGAGCACCTGGCTG

GCTGCAGGGTCCTACCTCTGGGTTTCCTGGAAGTGGATTTGGGCCAAGAAGGAGAGGGAG

AAAGGCCCGAGCCCCTGCCTTCCCGGGCCTTTCTCTCCTGGGCTGTCTCTGGTCTGGTCA

GCCTGGCTCTCGGGAAATTCAGCCATGAGCAGGGAAAGAACTCTCCCAACCCTGGGGCCT

AGCTGTATAGGAGGAATTGCCTAAGGGTGGCCCACTCTTGTGATTGCCCCATTTCCTCTG

GCAACAAAAGCCAGAGTGTTGTGGGCCAAGTCCCCCCACAGGGCCTCTGCAGGGCATGGC

CCTGATTTCCCTGGTTTGAGACTCACTTCCTCATCTCCCTGTCCTCTGAGATAATATGAG

TGAGCACTTAGGTATCATATCAGATGCTCAAGGCTGGCAGCTACCCCCTTCTTGAGAGTC

CAAGAACCTGGAGCAGAAATAATTTTTATGTATTTTTGGATTAATGAATGTTAAAAACAG

ACTCAGCTGTTTCTTTCCTTTTACTACTACCAGTTGCTCCCATGCTGCTCCACCAGGCCC

TGTTTCGGATGCCAACTGGCCCACTCCCCAAGCACTTGCCCCCAGCTTGCGACCATTGGC

ACTGGGAGGGCCTGGCTTCTGGGCTGATGGGTCAGTTGGGCCTTCATAAACACTCACCTG

GCTGGCTTTGCCTTCCAGGAGGAAGCTGGCTGAAGCAAGGGTGTGGAATTTTAAATGTGT

GCACAGTCTGGAAAACTGTCAGAATCAGTTTTCCCATAAAAGGGTGGGCTAGCATTGCAG

CTGCATTTGGGACCATTCAAATCTGTCACTCTCTTGTGTATATTCCTGTGCTATTAAATA

TATCAGGGCAGTGCATGTAAATCATCCTGATATATTTAATATATTTATTATATTGTCCCC

CGAGGTGGGGACAGTGAGTGAGTTCTCTTAGTCCCCCCAGAGCTGGTTGTTAAAGAGCCT

GGCACCTACCCGCTCTCACTTCATCTGTGTCATCTCTGCACACTCCAGCCCACTTTCTGC

CTTCAGCCATTGAGTGGAAGCTGCCCCAGGCCCTTACCAGGTGCAGATGCCCAATCTTGA

TGCCCAGCCATCAGAACTGTGAGCCAAATAAACCTTTTTCTGTATAAA

>NV14

GCCCGGCAGGTTGGCGGACCGGCGGGAGGCGCAGCCTGGGCAGAGCTCAGCTTGGTCCCG

CCGCCCGGCCGGTGCTCCCTGGCGCAGCCACGCAGGCGCACCGCAGACAGACCCCTCTGC

CATGAACCAGTCCATCCCAGTGGCTCCCACCCCACCCCGCCGCGTGCGGCTGAAGCCCTG

GCTGGTGGCCCAGGTGAACAGCTGCCAGTACCCAGGGCTTCAATGGGTCAACGGGGAAAA

GAAATTATTCTGCATCCCCTGGAGGCATGCCACAAGGCATGGTCCCAGCCAGGACGGAGA

TAACACCATCTTCAAGGCCTGGGCCAAGGAGACAGGGAAATACACCGAAGGCGTGGATGA

AGCCGATCCGGCCAAGTGGAAGGCCAACCTGCGCTGTGCCCTTAACAAGAGCCGGGACTT

CCGCCTCATCTACGACGGGCCCCGGGACATGCCACCTCAGCCCTACAAGATCTACGAGGT

CTGCTCCAATGGCCCTGCTCCCACAGACTCCCAGCCCCCTGAGGATTACTCTTTTGGTGC

AGGAGAGGAGGAGGAAGAAGAGGAAGAGCTGCAGAGGATGTTGCCAAGCCTGAGCCTCAC

AGAGGATGTCAAGTGGCCGCCCACTCTGCAGCCGCCCACTCTGCAGCCGCCCGTGGTGCT

GGGTCCCCCTGCTCCAGACCCCAGCCCCCTGGCTCCTCCCCCTGGCAACCCTGCTGGCTT

CAGGGAGCTTCTCTCTGAGGTCCTGGAGCCTGGGCCCCTGCCTGCCAGCCTGCCCCCTGC

AGGCGAACAGCTCCTGCCAGACCTGCTGATCAGCCCCCACATGCTGCCTCTGACCGACCT

GGAGATCAAGTTTCAGTACCGGGGGCGGCCACCCCGGGCCCTCACCATCAGCAACCCCCA

TGGCTGCCGGCTCTTCTACAGCCAGCTGGAGGCCACCCAGGAGCAGGTGGAACTCTTCGG

CCCCATAAGCCTGGAGCAAGTGCGCTTCCCCAGCCCTGAGGACATCCCCAGTGACAAGCA

GCGCTTCTACACGAACCAGCTGCTGGATGTCCTGGACCGCGGGCTCATCCTCCAGCTACA

GGGCCAGGACCTTTATGCCATCCGCCTGTGTCAGTGCAAGAGCTCATCCTGTTCCAAAAG

GGCCAGACCAACACCCCACCACCCTTCGAGATCTTCTTCTGCTTTGGGGAAGAATGGCCT

GACCGCAAACCCCGAGAGAAGAAGCTCATTACTGTACAGGTGGTGCCTGTAGCAGCTCGA

CTGCTGCTGGAGATGTTCTCAGGGGAGCTATCTTGGTCAGCTGATAGTATCCGGCTACAG

ATCTCAAACCCAGACCTCAAAGACCGCATGGTGGAGCAATTCAAGGAGCTCCATCACATC

TGGCAGTCCCAGCAGCGGTTGCAGCCTGTGGCCCAGGCCCCTCCTGGAGCAGGCCTTGGT

GTTGGCCAGGGGCCCTGGCCTATGCACCCAGCTGGCATGCAATAACAAGGCTGCAGACGG

TGACTGGCCCTGGCTTCCTGGGTGGCGGTGCGGACTGATGTGGAGATGTGACAGCCCCGA

TGAGCACCTGGCTGGCTGCAGGGTCCTACCTCTGGGTTTCCTGGAAGTGGATTTGGGCCA

AGAAGGAGAGGGAGAAAGGCCCGAGCCCCTGCCTTCCCGGGCCTTTCTCTCCTGGGCTGT

CTCTGGTCTGGTCAGCCTGGCTCTCGGGAAATTCAGCCATGAGCAGGGAAAGAACTCTCC

CAACCCTGGGGCCTAGCTGTATAGGAGGAATTGCCTAAGGGTGGCCCACTCTTGTGATTG

CCCCATTTCCTCTGGCAACAAAAGCCAGAGTGTTGTGGGCCAAGTCCCCCCACAGGGCCT

CTGCAGGGCATGGCCCTGATTTCCCTGGTTTGAGACTCACTTCCTCATCTCCCTGTCCTC

TGAGATAATATGAGTGAGCACTTAGGTATCATATCAGATGCTCAAGGCTGGCAGCTACCC

CCTTCTTGAGAGTCCAAGAACCTGGAGCAGAAATAATTTTTATGTATTTTTGGATTAATG

AATGTTAAAAACAGACTCAGCTGTTTCTTTCCTTTTACTACTACCAGTTGCTCCCATGCT

GCTCCACCAGGCCCTGTTTCGGATGCCAACTGGCCCACTCCCCAAGCACTTGCCCCCAGC

TTGCGACCATTGGCACTGGGAGGGCCTGGCTTCTGGGCTGATGGGTCAGTTGGGCCTTCA

TAAACACTCACCTGGCTGGCTTTGCCTTCCAGGAGGAAGCTGGCTGAAGCAAGGGTGTGG

AATTTTAAATGTGTGCACAGTCTGGAAAACTGTCAGAATCAGTTTTCCCATAAAAGGGTG

GGCTAGCATTGCAGCTGCATTTGGGACCATTCAAATCTGTCACTCTCTTGTGTATATTCC

TGTGCTATTAAATATATCAGGGCAGTGCATGTAAATCATCCTGATATATTTAATATATTT

ATTATATTGTCCCCCGAGGTGGGGACAGTGAGTGAGTTCTCTTAGTCCCCCCAGAGCTGG

TTGTTAAAGAGCCTGGCACCTACCCGCTCTCACTTCATCTGTGTCATCTCTGCACACTCC

AGCCCACTTTCTGCCTTCAGCCATTGAGTGGAAGCTGCCCCAGGCCCTTACCAGGTGCAG

ATGCCCAATCTTGATGCCCAGCCATCAGAACTGTGAGCCAAATAAACCTTTTTCTGTATA

AA
