# Supplementary material for: RNA-Seq for Enrichment and Analysis of IRF5 Transcript Expression in SLE
Source: PLoS One. 2013 Jan 18;8(1):e54487. doi: 10.1371/journal.pone.0054487 (PMC3548774; doi:10.1371/journal.pone.0054487)
Supplement: Dataset S2 — Sequences of the 19 junctions derived from the IRF5 variant transcriptome. Each junction is 94 bp in length and consists of 47-bp blocks of the two spliced exons at the area of intersection. (DOCX) [file pone.0054487.s008.docx]

**Supporting Dataset 2.** Sequences of the 19 junctions derived from the *IRF5* variant transcriptome. Each junction is 94 bp in length and consists of 47-bp blocks of the two spliced exons at the area of intersection.

>AA_ex2-ex3

CCACAAGGCATGGTCCCAGCCAGGACGGAGATAACACCATCTTCAAGGCCTGGGCCAAGG

AGACAGGGAAATACACCGAAGGCGTGGATGAAGC

>BB_ex2-ex6A

TCAACGGGGAAAAGAAATTATTCTGCATCCCCTGGAGGCATGCCACACTCTGCAGCCGCC

CACTCTGCAGCCGCCCGTGGTGCTGGGTCCCCCT

>CC_ex2-ex7

CACCCCGCCGCGTGCGGCTGAAGCCCTGGCTGGTGGCCCAGGTGAACACACGAACCAGCT

GCTGGATGTCCTGGACCGCGGGCTCATCCTCCAG

>DD_ex3-ex4

GCCCTACAAGATCTACGAGGTCTGCTCCAATGGCCCTGCTCCCACAGACTCCCAGCCCCC

TGAGGATTACTCTTTTGGTGCAGGAGAGGAGGAG

>EE_ex3del-ex4

ACGACGGGCCCCGGGACATGCCACCTCAGCCCTACAAGATCTACGAGACTCCCAGCCCCC

TGAGGATTACTCTTTTGGTGCAGGAGAGGAGGAG

>FF_ex4-ex5

AGGATTACTCTTTTGGTGCAGGAGAGGAGGAGGAAGAAGAGGAAGAGCTGCAGAGGATGT

TGCCAAGCCTGAGCCTCACAGGTGGGGCCGGGAG

>GG_ex4-ex6A

AGGATTACTCTTTTGGTGCAGGAGAGGAGGAGGAAGAAGAGGAAGAGATGCAGTGCAGTC

TGGCCCCCACATGACACCCTATTCTTTACTCAAA

>HH_ex4-ex6B

AGGATTACTCTTTTGGTGCAGGAGAGGAGGAGGAAGAAGAGGAAGAGAGGATGTCAAGTG

GCCGCCCACTCTGCAGCCGCCCACTCTGCGGCCG

>II_ex5-ex6A

GGTGTCCCTTCAGCTGCAGAGGATGTTGCCAAGCCTGAGCCTCACAGATGCAGTGCAGTC

TGGCCCCCACATGACACCCTATTCTTTACTCAAA

>JJ_ex5-ex6B

GGTGTCCCTTCAGCTGCAGAGGATGTTGCCAAGCCTGAGCCTCACAGAGGATGTCAAGTG

GCCGCCCACTCTGCAGCCGCCCACTCTGCGGCCG

>KK_ex6B-ex6D

TCAAAGAGGATGTCAAGTGGCCGCCCACTCTGCAGCCGCCCACTCTGCAGCCGCCCGTGG

TGCTGGGTCCCCCTGCTCCAGACCCCAGCCCCCT

>LL_ex6D-ex7

CGAACAGCTCCTGCCAGACCTGCTGATCAGCCCCCACATGCTGCCTCTGACCGACCTGGA

GATCAAGTTTCAGTACCGGGGGCGGCCACCCCGG

>MM_ex6intr-ex7

GTAAGGACCCATGGCTGGGCACGGGGAAGCAGTGCTGGGGGATTGGGTGACCGACCTGGA

GATCAAGTTTCAGTACCGGGGGCGGCCACCCCGG

>NN_ex7-ex8

GCGGGAGGTCAAGACCAAGCTTTTCAGCCTGGAGCATTTTCTCAATGAGCTCATCCTGTT

CCAAAAGGGCCAGACCAACACCCCACCACCCTTC

>OO_ex7-ex8del

GCGGGAGGTCAAGACCAAGCTTTTCAGCCTGGAGCATTTTCTCAATGGGGCCAGACCAAC

ACCCCACCACCCTTCGAGATCTTCTTCTGCTTTG

>PP_ex7del-ex8

AGCTACAGGGCCAGGACCTTTATGCCATCCGCCTGTGTCAGTGCAAGAGCTCATCCTGTT

CCAAAAGGGCCAGACCAACACCCCACCACCCTTC

>QQ_ex7p1-ex7p2

GCCAGCTGGAGGCCACCCAGGAGCAGGTGGAACTCTTCGGCCCCATATGCCATCCGCCTG

TGTCAGTGCAAGGTGTTCTGGAGCGGGCCTTGTG

>RR_ex8repeat

CAGCCTGGAGCATTTTCTCAATGAGCTCATCCTGTTCCAAAAGGGGTTCCAAAAGGGCCA

GACCAACACCCCACCACCCTTCGAGATCTTCTTCTG

>SS_ex8-ex9

AATGGCCTGACCGCAAACCCCGAGAGAAGAAGCTCATTACTGTACAGGTGGTGCCTGTAG

CAGCTCGACTGCTGCTGGAGATGTTCTCAGGGGA

>XX_ex1A-ex2

CCCGGCCGGTGCTCCCTGGCGCAGCCACGCAGGCGCACCGCAGACAGACCCCTCTGCCAT

GAACCAGTCCATCCCAGTGGCTCCCACCCCACCC
